# Fate and Efficacy of Engineered Allogeneic Stem Cells Targeting Cell Death and Proliferation Pathways in Primary and Brain Metastatic Lung Cancer

**DOI:** 10.1093/stcltm/szad033

**Published:** 2023-06-13

**Authors:** Susana Moleirinho, Yohei Kitamura, Paulo S G N Borges, Sophia Auduong, Seyda Kilic, David Deng, Nobuhiko Kanaya, David Kozono, Jing Zhou, Jeffrey J Gray, Esther Revai-Lechtich, Yanni Zhu, Khalid Shah

**Affiliations:** Center for Stem Cell and Translational Immunotherapy (CSTI), Brigham and Women’s Hospital, Harvard Medical School, Boston, MA, USA; Department of Neurosurgery, Brigham and Women’s Hospital, Harvard Medical School, Boston, MA, USA; Center for Stem Cell and Translational Immunotherapy (CSTI), Brigham and Women’s Hospital, Harvard Medical School, Boston, MA, USA; Department of Neurosurgery, Brigham and Women’s Hospital, Harvard Medical School, Boston, MA, USA; Center for Stem Cell and Translational Immunotherapy (CSTI), Brigham and Women’s Hospital, Harvard Medical School, Boston, MA, USA; Department of Neurosurgery, Brigham and Women’s Hospital, Harvard Medical School, Boston, MA, USA; Center for Stem Cell and Translational Immunotherapy (CSTI), Brigham and Women’s Hospital, Harvard Medical School, Boston, MA, USA; Department of Neurosurgery, Brigham and Women’s Hospital, Harvard Medical School, Boston, MA, USA; Center for Stem Cell and Translational Immunotherapy (CSTI), Brigham and Women’s Hospital, Harvard Medical School, Boston, MA, USA; Department of Neurosurgery, Brigham and Women’s Hospital, Harvard Medical School, Boston, MA, USA; Center for Stem Cell and Translational Immunotherapy (CSTI), Brigham and Women’s Hospital, Harvard Medical School, Boston, MA, USA; Department of Neurosurgery, Brigham and Women’s Hospital, Harvard Medical School, Boston, MA, USA; Center for Stem Cell and Translational Immunotherapy (CSTI), Brigham and Women’s Hospital, Harvard Medical School, Boston, MA, USA; Department of Neurosurgery, Brigham and Women’s Hospital, Harvard Medical School, Boston, MA, USA; Department of Radiation Oncology, Dana-Farber Cancer Institute, Harvard Medical School, Boston, MA, USA; Department of Chemical and Biomolecular Engineering, Johns Hopkins University, Baltimore, MA, USA; Department of Chemical and Biomolecular Engineering, Johns Hopkins University, Baltimore, MA, USA; Center for Stem Cell and Translational Immunotherapy (CSTI), Brigham and Women’s Hospital, Harvard Medical School, Boston, MA, USA; Department of Neurosurgery, Brigham and Women’s Hospital, Harvard Medical School, Boston, MA, USA; Center for Stem Cell and Translational Immunotherapy (CSTI), Brigham and Women’s Hospital, Harvard Medical School, Boston, MA, USA; Center for Stem Cell and Translational Immunotherapy (CSTI), Brigham and Women’s Hospital, Harvard Medical School, Boston, MA, USA; Department of Neurosurgery, Brigham and Women’s Hospital, Harvard Medical School, Boston, MA, USA; Harvard Stem Cell Institute, Harvard University, Cambridge, MA, USA

**Keywords:** lung cancer, NSCLC, E_V_DR_L_, leptomeningeal metastases, stem cells

## Abstract

Primary and metastatic lung cancer is a leading cause of cancer-related death and novel therapies are urgently needed. Epidermal growth factor receptor (EGFR) and death receptor (DR) 4/5 are both highly expressed in primary and metastatic non-small cell lung cancer (NSCLC); however, targeting these receptors individually has demonstrated limited therapeutic benefit in patients. In this study, we created and characterized diagnostic and therapeutic stem cells (SC), expressing EGFR-targeted nanobody (E_V_) fused to the extracellular domain of death DR4/5 ligand (DR_L_) (E_V_DR_L_) that simultaneously targets EGFR and DR4/5, in primary and metastatic NSCLC tumor models. We show that E_V_DR_L_ targets both cell surface receptors, and induces caspase-mediated apoptosis in a broad spectrum of NSCLC cell lines. Utilizing real-time dual imaging and correlative immunohistochemistry, we show that allogeneic SCs home to tumors and when engineered to express E_V_DR_L_, alleviate tumor burden and significantly increase survival in primary and brain metastatic NSCLC. This study reports mechanistic insights into simultaneous targeting of EGFR- and DR4/5 in lung tumors and presents a promising approach for translation into the clinical setting.

## Graphical Abstract

**Graphical Abstract:**
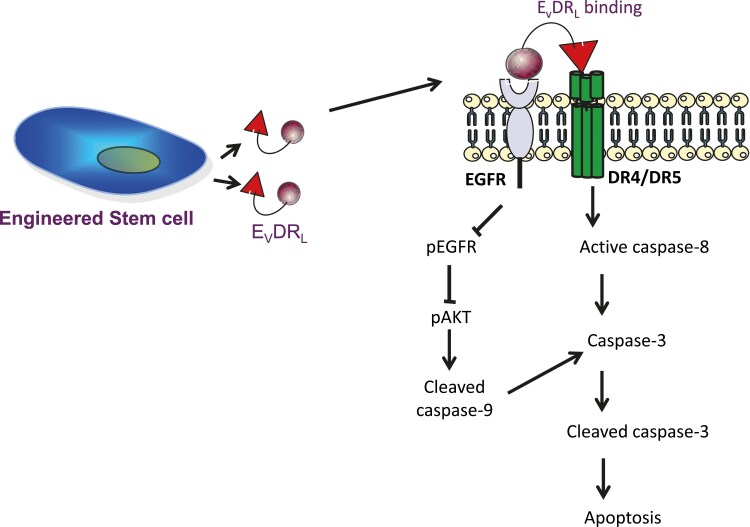

**Significance Statement**

This study provides a platform for developing stem cell-based targeted therapies in NSCLC and associated advanced leptomeningeal metastases. Given that the overall 5-year relative survival rate for lung cancer patients is very low, this study provides the rationale for the potential application of therapies that simultaneously target EGFR and DR4/5 in the treatment of lung neoplasias.

## Introduction

Lung cancer is a major health problem worldwide and is the leading cause of cancer-related death for both men and women. Although several attempts have been made to develop effective treatment strategies, the overall 5-year relative survival rate remains at 18.^1^ Histological subtype heterogeneity, limited understanding of tumor biology, relatively late disease presentation with metastasis in distant organs, and development of drug resistance are the main causes of poor diagnosis. Advanced-stage NSCLC (corresponding to the overwhelming majority of cases) is developed to an extent that precludes surgical resection and as such is treated with chemotherapy, targeted drugs, or immunotherapy.^2^ Among the advanced stage NSCLC, leptomeningeal metastasis (LM), a type of brain metastasis formed from the dissemination of cancer cells into the cerebrospinal fluid (CSF) compartment, is the most devastating condition with an average survival of around 3-4 months post-diagnosis.^3,4^ Molecularly driven approaches represent the major treatment options for patients with NSCLC, due to the identification of driver oncogene mutations in a reasonable percentage of patients (approximately 15% in Caucasian population).^5-7^

The deregulated expression of epidermal growth factor (EGFR) through mutation or amplification is commonly found in NSCLC and NSCLC-LM. Small molecule EGFR tyrosine kinase inhibitors (TKIs) directed against this receptor, such as gefitinib, erlotinib, and afatinib, have changed the paradigm of care for NSCLC, particularly in patients harboring activating EGFR mutations.^8^ Although treatment with TKIs shows superior efficacy compared with traditional chemotherapies in managing LM,^9-11^ development of resistance to these small molecules is a frequent feature of disease progression. This represents an important limitation to its efficiency as LM develops at late stages of NSCLC when patients have already acquired resistance to EGFR-TKIs.^8,9^ This highlights the urgent need to uncover alternative therapies, especially for the primary-site disease.

Targeting apoptotic machinery has become an attractive therapeutic approach for primary and metastatic NSCLC.^12,13^ Strategies that selectively trigger apoptosis in tumor cells include targeting the tumor necrosis factor (TNF)-related apoptosis-inducing ligand (TRAIL) receptors—death receptors 4 and 5 (DR4/5). Both receptors have been shown to be overexpressed at the cell membrane in NSCLC samples compared to normal lung epithelium,^14^ and to act synergistically with a range of chemotherapeutic drugs such as cisplatin and pemetrexed,^15-18^ providing a rationale for the use of DR4/5 targeted therapy in NSCLC. Due to promising preclinical results, several clinical trials have been conducted in NSCLC with agonist antibodies against DR4/5 or recombinant Apo2L/TRAIL agents. Some of these studies have also evaluated their efficacy in metastatic NSCLC patients including BM^13^ (clinicaltrials.gov).

The development and application of bi-functional therapeutic agents and alternative delivery methodologies overcoming the limitations of current receptor-targeted therapies offers an effective approach to the treatment of NSCLC. Antibody-based therapies have been widely explored through the development of monoclonal antibodies (mAbs) technology^19,20^; however, this treatment modality still possesses notable limitations such as non-homogenous distribution of mAb at the tumor site, potentially leading to the survival of subpopulations of cancer cells and concomitant tumor relapse.^21,22^ To bypass this limitation, engineering approaches using functional antibody fragments such as antigen-binding fragments (Fab) or single-chain variable fragments (scFv) have been used in various clinical settings with relative success and already in clinical trials.^23-25^ Although smaller than mAbs, their size still limits homogenous distribution within the tumor (Fab ~50 kDa; scFv, ~28 kDa), and non-ideal binding affinity. Single domain antibodies, or nanobodies, are antibody fragments that, although smaller than Fabs or scFv, are still able to selectively bind an antigen. These fragments, originating from *Camelid* heavy-chain antibodies (VHH, ~15 kDa),^26^ exhibit appealing characteristics for in vivo applications such as small size, high specificity, affinity, solubility, and stability. We and others have already developed and characterized therapeutic nanobodies against cancer-related extracellular targets such as EGFR, HER2, c-Met, VEGFR, and DR5.^27,28^

Concerning the efficient delivery of the therapy to the tumor site in these neoplasias, intravenous infusion results in a relatively low concentration of therapeutic agents at the tumor site.^29,30^ While this could potentially be overcome by increasing the initial drug concentration this would increase systemic toxicity and adversely affect the overall life quality of the patient.^31^ Several studies conducted by us and others have shown that gene-modified mesenchymal stem cells (MSCs) represent an attractive candidate for delivery of cell-based therapies.^32-35^ Genetically engineered “off the shelf” allogeneic MSCs specifically home to and reside at the tumor site, where they deliver a constant dose of therapy. Thus, this approach can overcome an important limitation of intravenous drug delivery in lung cancer patients.

In this study, we developed an optimized secretable bi-functional molecule, E_V_DR_L,_ consisting of EGFR specific VHH (E_V_) fused to the extracellular domain of DR ligand (DR_L_) via a linker sequence and an isoleucine zipper and extensively characterized its functionality in a broad spectrum of NSCLC and NSCLC-LM cell lines. We first tested the homing and biodistribution of intravenously (I.V.) delivered MSC and then tested the efficacy of MSCs-delivered E_V_DR_L_ in NSCLC and NSCLC-LM tumor models.

## Material and Methods

### Animal Studies

All animal experiments were performed in accordance with a protocol approved by Brigham and Women’s Hospital Institutional Animal Care and Use Committee. Female athymic nu/nu (Envigo) and NOD/SCID (Charles River Laboratories) 4-6 weeks of age were used for all in vivo experiments.

### Orthotopic Thoracic Implantation

Orthotopic thoracic implantation of NSCLC was carried out as previously described.^36^ Briefly, SW900-FmC cell cultures were harvested, and single-cell suspensions of >90% viability with the indicated number of cells resuspended in 35 µL of 1× PBS. Cells were then mixed with matrigel (Corning) at 1:1 ratio and kept on ice until injection. Anesthetized mice were placed in the lateral decubitus position with the left chest facing up and a small (0.5-1 cm) incision was made over the skin just below the scapula. The chest wall muscles (connective tissue) were gently spread until the intercostal space and thoracic ribs were clearly visible and the left lobe of the lung evident. The site of tumor injection was determined by counting from the lower border of the rib cage upward, between the 6th and the 7th rib at the posterior axillary line. At this location, the syringe was gently introduced until its tip touched the intercostal space followed by 3.5 mm penetration into the lung parenchyma and the tumor cell mixture was injected. After injection, the syringe was gently removed and the incision site closed. Mice were allowed to recover in a preheat blanket for 20-30 min and subsequently given analgesic medication. All in vivo procedures were approved by the Subcommittee on Research Animal Care at Brigham and Women’s Hospital (BWH).

### Leptomeningeal Metastasis Model

Intrathecal administration was conducted as previously reported.^35^ Briefly, female nude mice (6-8 weeks of age) were anesthetized and after immobilization on a surgical platform, the dura matter located between the skull and atlas vertebra was exposed. PC9 BrM3-GFP-Fluc (3 × 10^4^ cells per mouse) or SW 900 BM-Fluc-Mcherry (4 × 10^4^ cells per mouse) was slowly intracisternally inoculated through a catheter connected to a Hamilton microsyringe. After inoculation, the cathether was gently removed and the hole in the dura matter was immediately closed with a piece of occipital muscle. For analysis of the therapeutic efficacy of E_V_DR_L_, hMSC-GFP or hMSC-E_v_DR_L_ (5 × 10^5^ cells per mouse) was injected through the same hole in a similar form as tumor cells.

### Clinical Samples and Immunohistochemistry

NSCLC patient samples were obtained from the Brigham Women’s Hospital. All samples were in compliance with protocols approved by Institutional review Board (IRB). The paraffin sections were deparaffinized and rehydrated followed by antigen retrieval with sodium citrate buffer (pH 6). After, endogenous peroxidase was quenched using 3%H_2_O_2_ in methanol for 30 min at room temperature. After 2 washes with distilled water sections were blocked for 1 h in PBS containing 1% BSA, 0.1% Tween 20, and 5% normal goat serum in a humidified chamber at room temperature. Sections were then incubated with indicated primary antibodies diluted in PBS supplemented with 1% BSA and 0.1% Tween 20 overnight at 4 °C. The following day, sections were extensively washed in PBS containing 1% BSA, 0.1% Tween 20, and incubated with goat anti-rabbit HRP polymer (1:1; [Abcam]) for 2 h at room temperature. Samples were washed thrice, color developed, and counterstained with hematoxylin. Finally, the sections were dehydrated, cleared, and mounted with xylene-based mounting medium for microscope evaluation (Cytoseal XYL, Thermo Fisher Scientific).

### Statistical Analysis

Data were analyzed by applying an unpaired, 2-tailed Student’s *t*-test when comparing 2 experimental groups and expressed as mean ± S.D for in vitro analysis (except when stated otherwise) and as mean ± S.E.M. for BLI imaging analysis of tumor volumes. Differences were considered statistically significant as follows: *P < .05; **P < .01; ***P < .001; n.s.—non-significant. Kaplan-Meier survival curves were generated using Prism 5 software (GraphPad Software) and related *P*-values were obtained with log-rank (Mantel-Cox) 2-sided tests.

## Results

### EGFR and Death Receptors (DR4/5) are Suitable Therapeutic Targets in NSCLC

**Figure 1.**
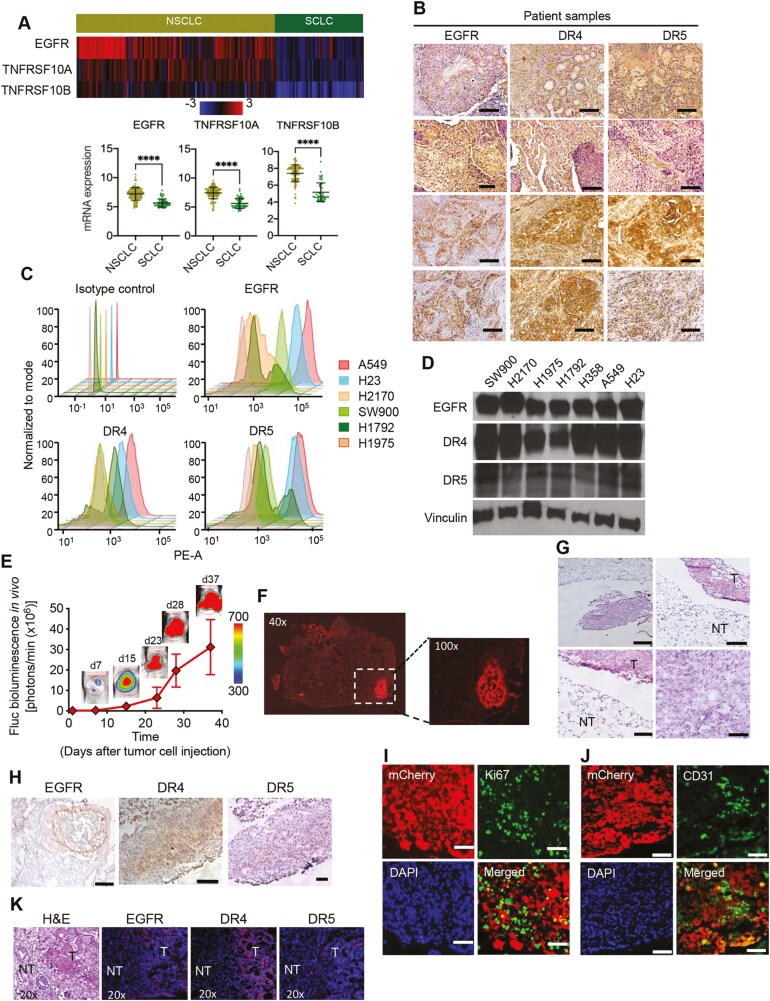
EGFR, DR4, and DR5 show upregulated expression in NSCLC tissue patients and mouse tumor models. (**A**) Top: heatmap showing the relative transcriptional expression level of *EGFR*, *TNFRSF10A*/*B* (DR4/5) in patient samples of NSCLC versus SCLC (data obtained from TCGA RNA-seq. database) (*n* = 1144 samples). Bottom: Comparison of *EGFR*, *DR4*, and *DR5* mRNA levels between lung cancer subtypes (*n* = 174 samples). **(B)** Immunohistochemistry images of NSCLC patient tissue samples stained with anti-EGFR, anti-DR4, and anti-DR5 antibodies (*n* = 4). Pictures show representative fields at 200× magnification. Scale bar = 100 μm. **(C)** FACS analysis of the expression of cell surface markers EGFR, DR4, and DR5 in SW900, H2170 (lung squamous carcinoma cell lines), A549, H23, H1792, and H1975 (lung adenocarcinoma cell lines). Histograms correspond to the medium fluorescence intensity of MAb staining normalized to mode. Analyses conducted twice in duplicate (*n* = 4). **(D)** Screening of the expression of whole protein EGFR, DR4, and DR5 in the cell lines specified as determined by Western blotting. Vinculin was used as loading control. The blots shown are representative of 3 independent experiments. **(E)** In vivo plot of bioluminescence imaging showing tumor growth and its quantification in immunocompromised NOD/SCID or athymic nude mice bearing SW900-FmC (0.5 × 10^6^ cells) (*n* = 5 animals). **(F)** Representative photomicrographs (fluorescence) of 8-10 μm thicknesses coronal lung sections bearing an established SW900-FmC tumor. Fluorescence micrographs show representative fields at 40× and 100× magnification (tumor detailed). **(G)** H&E staining of coronal mouse lung tumor sections described in (F). Scale bar = 500 μm. T, tumor area; NT, non-tumor area. **(H)** Immunohistochemistry of mouse coronal lung tumor sections stained with anti-EGFR, anti-DR4, and anti-DR5 antibodies. Pictures show representative fields. Scale bar = 100 μm. **(I)** Coronal mouse lung tumor sections described in (F) were subjected to immunofluorescence staining with anti-Ki67 and **(J)** anti-CD31 antibodies. DAPI was used for nuclei fluorescence staining. Pictures show representative fields at 200× magnification. Scale bar = 100 μm. (**K)** Representative microphotographs of coronal mouse lung tumor sections described in (F) showing H&E and immunofluorescence staining with anti-EGFR, anti-DR4, and anti-DR5 antibodies. Pictures show representative fields at 20× magnification. T, tumor area; NT, non-tumor area.

To determine the expression levels of *EGFR* and *DR4/5* (*TNFRSF10A/B*) in lung cancers, we analyzed their transcriptional levels in the major 2 histological sub-types NSCLC and SCLC (small cell lung cancer; cancer cell line encyclopedia-TCGA database). All the 3 cell surface receptors showed significantly higher RNA levels in NSCLC than in SCLC (Fig. 1A). On the basis of these findings, NSCLC was chosen as the most suitable histological type to further investigate the therapeutic potential of cell surface-directed therapies. Initially, we confirmed the upregulated expression of EGFR and DR4/5, at the protein level by immunohistochemistry of tissue sections from an NSCLC patient compared with sections of a healthy/normal lung (Fig. 1B; Supplementary Supplementary Fig. S1). We then screened a panel of established cell lines of both adenocarcinomas and squamous cell carcinomas for the endogenous expression of these cell surface receptors. Fluorescence-activated cell sorting (FACS) and Western blotting showed varying expression levels both at the cell surface and intracellularly in A549, H23, H1792, H1975 (adenocarcinoma), and SW900 and H2170 (squamous cell carcinoma) cell lines (Fig. 1C, 1D).

Next, to recapitulate the clinical setting observed in patients, we orthotopically implanted human cell line SW900 engineered to express a bimodal protein mCherry-firefly luciferase (Fluc) (FmC) in left lateral thorax to develop imageable mouse models of NSCLC in immunocompromised SCID or athymic nude mice^36^ (Supplementary Fig. S2A). Noninvasive bioluminescence imaging (BLI) showed a gradual increase of Fluc signal in SW900-FmC bearing mice (Fig 1E). Around day 37, the cohort showed an average weight loss of approximately 20% from baseline (Supplementary Fig. S2B) and animals showed signs of moribundity due to their significant tumor burden (Supplementary Fig. S2C, S2D). Phase-contrast microscopy of coronal sections from tumor-bearing mice showed the development of neoplastic lesions of different sizes and distributed across the lung lobules (Fig. 1F). Histopathological analysis on lung sections by H&E staining confirmed the presence of tumors, and IHC and immunofluorescence staining with EGFR- and DR4/5-specific antibodies depicted the upregulation of these markers in the NSCLC in vivo (Fig. 1G, 1H, 1K). Further, we observed an increased expression of Ki67 and CD31 in tumor cells in histologic lung sections (Fig. 1I, 1J). Together, these results show that EGFR and DR4/5 are upregulated in NSCLC both in patient samples and in vivo mouse NSCLC models, and thus represent suitable candidates to investigate the therapeutic potential of therapies targeted at these receptors.

### E_V_DR_L_ is Highly Effective in Inducing Cell Death in a Broad Spectrum of NSCLC by Inhibition of EGFR Signaling and Activation of Apoptosis-Mediated Pathways

**Figure 2.**
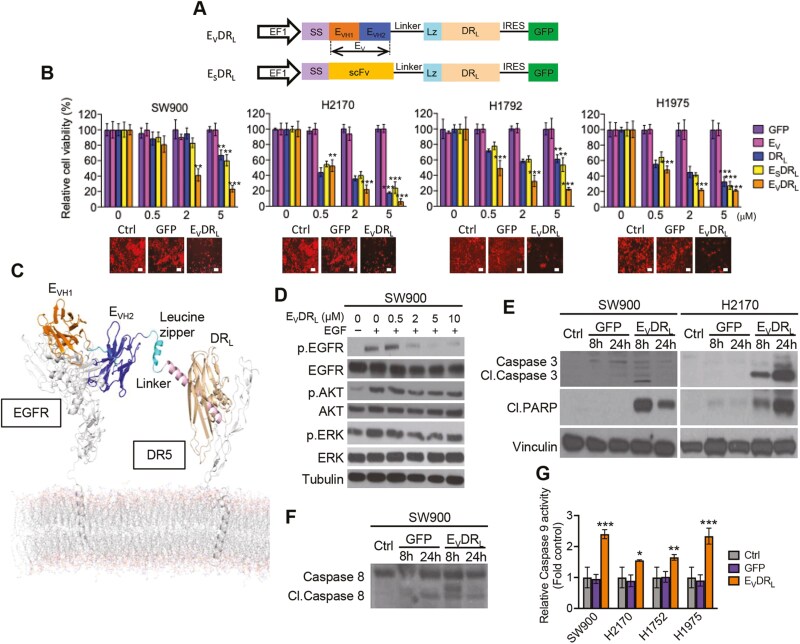
E_V_DR_L_ activates main downstream effectors of EGFR and DR4/5 pathways and inhibits viability of NSCLC. **(A)** Schematic representation of lentiviral transfer vectors composed by either anti-EGFR nanobody or anti-EGFR single-chain variable fragment (scFv) antibody domains. SS, signal sequence. **(B)** SW900, H2170, H1792, and H1975 cell lines cells stably transduced with lentiviral vectors encoding *Firefly* luciferase—mCherry (FmC) were treated with different concentrations (0, 0.5, 2, and 5 μM) of GFP, E_V_, DR_L_, E_S_DR_L_, or E_V_DR_L_, for 72 h and cell viability was determined. Means were calculated from 3 independent experiments conducted in triplicate (*n* = 9). Error bars represent ± S.D. **P < .01; ***P < .001 relative to the control GFP. Scale bar = 100 μm. Phase-contrast pictures show prominent loss of cell viability after E_V_DR_L_ treatment (5 μM for 72 h) compared with control treatments at the same conditions or untreated cell lines. **(C)** Model of EGFR-E_V_-DR_L_-DR5 complex embedded in a membrane bilayer showing the binding of E_V_ domain (consisting of E_VH1_ and E_VH2_) binds to 2 different regions of EGFR and DR_L_ binds to DR4/5. **(D)** Western blotting analysis on lysates obtained from SW900 cell lines treated with Epidermal growth factor (EGF) (20 ng/mL) for 5 min and E_V_DR_L_ for 24 h (0.5, 2, 5, and 10 μM respectively) after 4 h serum starvation. Tubulin and vinculin were used as loading controls. **(E)** SW900 and H2170 cell lines were treated either with GFP or E_V_DR_L_ for 8 or 24 h. Cell lysates were collected and Western blotting was performed for determination of cleaved caspase 3 and PARP expression levels compared with untreated cells used as a control. Vinculin was used as a loading control. (**F**) SW900 cell line was treated either with GFP or E_V_DR_L_ for 8 or 24 h. Cell lysates were collected and Western blotting was performed for determination of caspase 8 expression levels compared with untreated cells, used as a control. Vinculin was used as a loading control. **(G)** Cells were treated for 24 h with E_V_DR_L_ and caspase 9 expression levels determined by Cell Titer Glo. SW900 and H2170 cell lines showed more pronounced upregulation of caspase 9 after treatment than H1792 or H1975 compared with GFP-treated cells used as control. Means were calculated from 2 independent experiments conducted in triplicate (*n* = 6). Error bars represent ± S.D. *P < .05; **P < .01; ***P < .001.

To simultaneously target EGFR-mediated cell proliferation and DR4/5-mediated death pathways we engineered 2 bi-functional proteins, consisting of cDNA fusions encoding VHH domain of EGFR blocking nanobody (E_V_) or single-chain fragment variable (scFv) of EGFR (E_S_) and a cytotoxic extracellular domain of TRAIL (DR_L_) fused to a linker sequence and a leucine zipper domain (Fig. 2A). These fusions were cloned in front of the EF1 promoter in lentiviral transfer vector, packaged and the resulting virus; LV-E_V_DR_L_ and LV-E_S_DR_L_ were used to transduce HEK293T cells. Conditioned medium from LV-E_V_DR_L_ transduced HEK293T cells resulted in a significant cell death in a cohort of lung tumor cells as compared to the E_V_, DR_L_, and E_S_DR_L_. All analyzed cell lines showed higher sensitivity to treatment with E_V_DR_L_ at the indicated concentrations and after 72 h compared to controls (Fig. 2B; Supplementary Figs. S3A–S3D, S4). E_V_DR_L_ bi-functional protein is a modified version of the original fusion protein ENb-TRAIL^27,37^ and lacks Flt3 extracellular domain.

The excision of Flt3 domain from the original molecule was hypothesized to promote maximum linking between EGFR and DR4/5 by avoiding binding of Flt3L to its receptor FLT3, and therefore to enhance molecule’s efficacy. We compared the efficiency of both E_V_DR_L_ and ENb-TRAIL and found that E_V_DR_L_ induces higher sensitivity to treatment and subsequent cell death compared with controls (Supplementary Fig. S5). Protein modeling studies confirmed the binding of E_V_ domain (consisting of E_VH1_ and E_VH2_) to 2 different regions of EGFR and the binding of DR_L_ to DR4/5 (Fig. 2C).

EGF competition studies and subsequent Western blotting analysis showed that E_V_DR_L_ specifically binds to EGFR and competes with EGF ligand binding to EGFR. This resulted in inhibition of EGFR signaling (Fig. 2D). Specifically, higher concentrations of E_V_DR_L_ induced the downregulation of phosphorylated EGFR and its downstream components AKT and extracellular regulated kinase (ERK) in SW900 cell line. Next, we investigated whether E_V_DR_L_ activates the apoptotic machinery by binding to DR4/5. Treatment with 2µM E_V_DR_L_ activated the extrinsic apoptotic pathway, as determined by increased expression of cleaved caspase 3 and PARP in both SW900 and H2170 cell lines compared to the control (Fig. 2E). Caspase 8 also displayed an upregulated expression upon E_V_DR_L_ treatment after 8h in SW900 cells (Fig. 2F). The relative activity of caspase 9 was particularly high in SW900 and H1975 cells, with a moderate increase in the H2170 and H1792 cell lines, 24 h after E_V_DR_L_ treatment (Fig. 2G). Analysis of the relative caspase 3/7 activity also showed increased expression upon treatment with E_V_DR_L_ on SW900 and H2170 cell lines (Supplementary S6A, S6B). Collectively, these results reveal that the robust killing of NSCLC cell lines is mediated by inhibition of the pro-proliferative EGFR pathway combined with DR4/5-triggered caspase-induced apoptosis.

### Binding of E_V_DR_L_—E_V_ Domain to EGFR is Critical for E_V_DR_L_ Full Activation and Triggering of Apoptotic-Mediated Pathways

**Figure 3.**
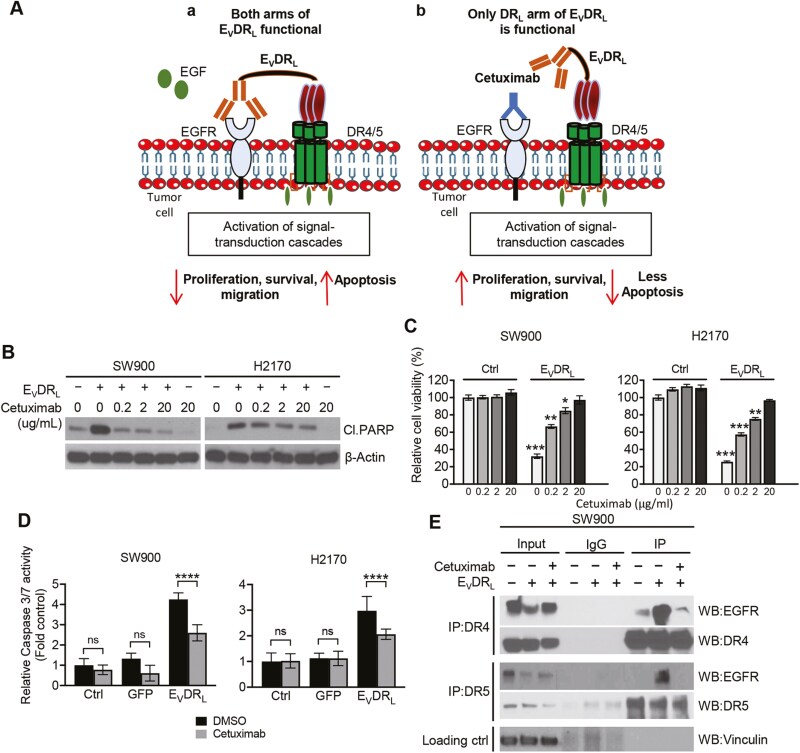
E_V_ domain needs to bind EGFR for full downstream activation of E_V_DR_L_ (**A**) Schematic representation showing E_V_DR_L_ mechanism of action (A) and cetuximab binding to EGFR blocking downstream EGFR signaling pathway and partially inhibiting E_V_DR_L_ induction of apoptosis (B). **(B)** Western blot analysis showing PARP cleavage on lysates from SW900 and H2170 cell lines incubated with 0, 0.2, 2, or 20 ug/mL cetuximab for 30 min followed by treatment with E_V_DR_L_ for 24 h (5 μM). **(C)** Cell viability analysis of H2170 and SW900 cell lines treated with the same conditions described in (B). Means were calculated from 3 independent experiments conducted in triplicate (*n* = 9). Error bars represent ± S.D. *P < .05; **P < .01; ***P < .001. **(D)** Relative caspase 3/7 activity analysis on H2170 and SW900 cell lines treated with either cetuximab or DMSO (20 μg/mL) for 30 min, followed by treatment with either GFP or E_V_DR_L_ (5 μM) for 8 h. Means were calculated from 3 independent experiments conducted in triplicate (*n* = 9). Error bars represent ± S.D. *P < .05; **P < .01; ***P < .001. **(E)** SW900 cell line was treated with GFP, E_V_DR_L_ or E_V_DR_L_ + cetuximab (20 μg/mL). Total cell lysates (Input) and DR4 or DR5 immunoprecipitates (IP) were subjected to immunoblotting analysis with anti-EGFR, anti-DR4, or anti-DR5 antibodies as indicated. Vinculin was used as loading control. IgG was used as a non-specific antibody control for IPs throughout. The blots shown are representative of 2 independent experiments.

To verify the specificity of E_V_DR_L_ toward EGFR, we treated SW900 and H2170 cell lines with cetuximab, a recombinant human/mouse chimeric EGFR monoclonal antibody.^38^ Both cetuximab and EGFR nanobody target the extracellular domain III of EGFR and therefore cetuximab should block E_V_DR_L_ binding to EGFR^39-41^ (Fig. 3A). Cetuximab treatment showed no induction of cell death when used as a mono-therapeutic agent or co-treatment with control (Fig. 3B, 3C). However, combination of cetuximab and E_V_DR_L_ (2 µM) resulted in reduced cleaved PARP and caspase 3/7 activities. It also decreased the cell lines susceptibility to E_V_DR_L_ treatment as observed by treating SW900 and H2170 with increasing concentrations of the anti-EGFR mAb (Fig. 3B–3D; Supplementary Fig. S6C). As expected, the decreased rather than total abolishment of cytotoxic effect is due to the intact apoptotic machinery triggered by the DR_L_ arm. Next, we determined whether the striking effect observed in NSCLC cell lines following treatment with E_V_DR_L_ relies on the formation of a complex between DR4/5 and EGFR. Co-immunoprecipitation (co-IP) analyses using anti-DR4 or anti-DR5 antibodies revealed an association between DR4/5 and EGFR (Fig. 3E). This association was abolished by the addition of cetuximab. Therefore, our results showed that E_V_DR_L_ fusion protein binds to EGFR and DR4/5 and results in the formation of the complex between DRs and EGFR which is critical for inhibition of EGFR pro-proliferative pathway and activation of the apoptotic cascade.

### Allogeneic MSC Home to the Tumors in the Lung

**Figure 4.**
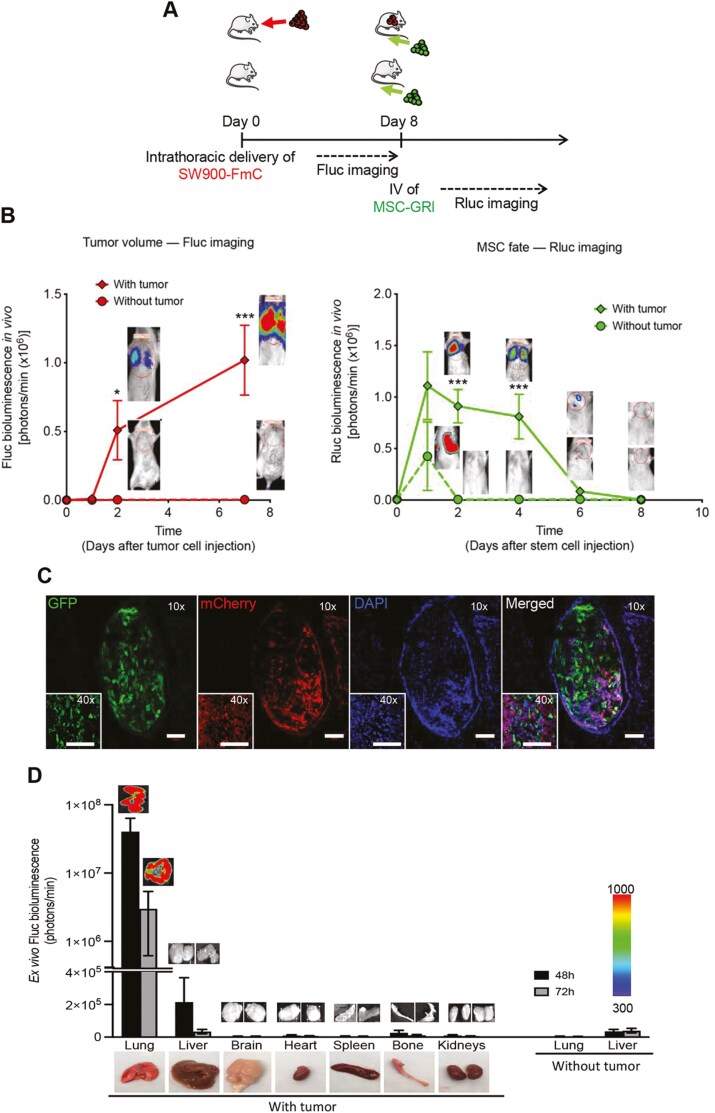
MSCs home to NSCLC in vivo. (**A**) Schematic of the experimental design. Mice bearing SW900-FmC in the left lobe of the lung were injected with MSC-Rluc-GFP cells (2 × 106 cells/200 μL) via tail vein and the presence of MSCs monitored by BLI of *Renilla* luciferase signal 2 and 4 days after implantation. **(B)** Plots and dual bioluminescence imaging showing the progression of tumor volumes (Fluc imaging) and MSC fate (Rluc imaging) in lung tumor bearing and non-tumor bearing mice (*n* = 3 animals per condition). Error bars represent ±S.D. *P < .05; ***P < .001. **(C)** Representative photomicrographs (fluorescence) of coronal lung sections show tropism of GFP-labeled stem cells toward SW900-FmC tumor cells. DAPI was used for nuclei fluorescence staining. Fluorescence confocal imaging show representative fields at 100×, 200×, and 400× magnification. Scale bar = 100 μm. **(D)** Biodistribution analysis of MSC-fate after I.V. implantation in tumor and non-tumor bearing lungs over 72 h. Ex vivo Rluc analysis of different organs collected individually 48 h and 72 h after tail injection shows predominant presence of MSC-GFP-Rluc in the lungs followed by the liver in tumor-bearing mice. This is not observed in non-tumor bearing lungs. The remainder analyzed organs specifically brain, heart, spleen, bone, and kidneys denoted residual/background signal (*n* = 2 animals per condition).

To determine whether MSCs could represent a viable delivery methodology in our mouse tumor models, we initially tested the ability of MSCs to home to and reside at the lung tumor site (Fig. 4A). Non-tumor bearing or NOD/SCID mice bearing SW900-FmC tumors were intravenously injected with bone marrow-derived mouse MSC engineered to express a bimodal imaging marker, GFP-Renilla luciferase (Rluc) (GRl) (Supplementary Fig. S7A–S7C). MSC-GRl cells were detected in the lungs of tumor-bearing mice on days 2 and 4 (Fig. 4B) in all the implanted mice; however, by day 6, only 1 animal still showed the presence of MSCs as indicated by BLI of Rluc signal, indicating the necessity to systemic delivery of these cells to improve the efficiency of MSC-induced therapy.

In tumor-free mice, with the exception of day 1 post-administration, BLI of Rluc showed no signal in the lungs, suggesting increased difficulty for cell engraftment in the absence of lung tumors (Fig. 4B). To further confirm the homing of MSCs to the tumor site, the lungs of tumor-bearing mice were harvested 2 days after intravenously delivery of MSC-GRl. Confocal imaging on histological sections showed GFP-labeled MSCs located either peritumorally or at the mCherry-labeled tumor cells (Fig. 4C). To assess the in vivo distribution of intravenously delivered MSCs, mice bearing established orthothopic SW900-mCherry-Rluc (-RmC) lung tumors were injected intravenously with MSCs-GFP-Fluc (-GFl). Ex vivo Fluc analysis of various organs harvested at the indicated time points showed strongest presence of MSCs in the lung tumors over a period of 72 h (Fig. 4D). In non-tumor bearing mice, no MSC presence was observed in the lungs at either 48 or 72h (Fig. 4D) In the remaining organs (brain, heart, spleen, bone, and kidneys), MSCs were not detected at any of the analyzed time points in either tumor-bearing or non-tumor bearing animals. Together these results suggest that intravenously delivered MSCs home to the tumor loci in the lungs and engraft at this location for at least a period of 72 h, suggesting that this methodology can be effective for anti-tumor therapy delivery in NSCLC.

### MSC-E_V_DR_L_ Suppresses Tumor Growth and Extends Survival of Mice Bearing NSCLC

**Figure 5.**
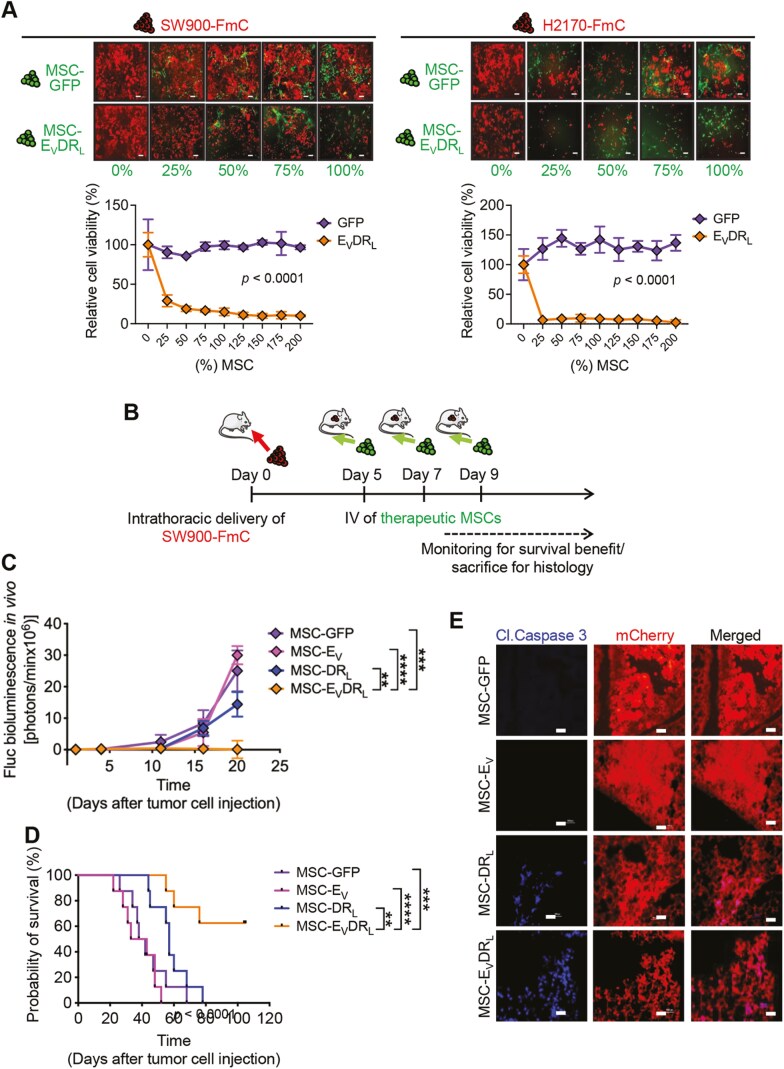
MSCs expressing E_V_DR_L_ efficiently inhibit tumor proliferation in vitro and shrink lung tumor volume in vivo. (**A**) Representative photomicrographs (top) and assessment of viability (bottom) of SW900-FmC or H2170-FmC cocultured with increasing percentages (0%-200%, as indicated on the x-axis) of MSC-E_V_DR_L_ or GFP only. Means were calculated from 3 independent experiments conducted in triplicate (*n* = 9). Error bars represent ±S.D. ***P < .001 relative to GFP treatment control and in all analyzed concentrations. Pictures show representative fields at 200× magnification. Scale bar = 100 μm. **(B)** Schematic of the in vivo therapeutic assessment experimental design. **(C)** Plot showing changes in tumor volumes from each treatment group before and after MSCs implantation (pre-treatment—days 1 and 4; post-treatment—days 11, 16, and 20) (*n* = 8 animals). Error bars represent ±S.E.M. **P < .01; ***P < .001 **(D)** Kaplan-Meier survival curves of athymic nude mice bearing SW900-FmC tumors I.V. injected with the indicated therapeutic groups as described in (B). *P* values by Mantel-Cox (log-rank) test, ***P < .001 (MSC-E_V_DR_L_), **P < .01 (MSC-DR_L_), n.s.—non-significant (MSC-E_V_); all relative to GFP treatment control. **(E)** Immunofluorescence staining with anti-cleaved caspase 3 antibodies on coronal mouse lung tumor sections (8—10 μm thicknesses) of each one of the analyzed therapeutic groups described in (B). For E, mice were sacrificed 20 days after tumor injection corresponding to day 11 following systemic delivery of therapies. Pictures show representative fields at 200× magnification. Scale bar = 100 μm.

Next, we sought to determine the direct antitumor effect of MSC-E_V_DR_L_ in vitro and in the orthotopic xenograft mouse model of NSCLC. Human MSC (hMSC) and mouse MSC were engineered to express E_V_DR_L_ by transducing MSC with LV-E_V_DR_L_-IRES-GFP. hMSC released E_V_DR_L_ in a conditioned medium (Supplementary Fig. S8A) and resulted in a significant reduction of cell viability when cocultured in different ratios with either SW900-FmC or H2170-FmC at 72 h (Fig. 5A; Supplementary Fig. S8B). In vivo, immunodeficient athymic nude mice bearing SW900-FmC tumors in the lungs were intravenously treated with MSC-E_V_DR_L_, MSC-DR_L_, MSC-E_V_, or MSC-GFP (Fig. 5B; Supplementary Fig. S9). The tumor growth was substantially suppressed as early as day 11 following systemic delivery of MSC-E_V_DR_L_ as compared with controls, which continued to exhibit robust tumor development (Fig. 5C; Supplementary Fig. S10A. S10B). Consistent with our previous observations, tumors treated with MSC-E_V_DR_L_ showed slower tumor growth compared with MSC-GFP or MSC-E_V_ only therapies. This effect translated into a significantly prolonged survival benefit in mice treated with MSC-E_V_DR_L_ with 62.5% of the treated mice (5 out of 8) surviving beyond 100 days (vs. median survivals of MSC-GFP: 37.5 days; MSC-E_V_: 32 days; MSC-DR_L_: 56 days—*P*-values < .0001) (Fig. 5D). Furthermore, examination of the expression of cleaved caspase 3 showed upregulated expression of the apoptotic marker in the lung sections treated with MSC-E_V_DR_L_ compared with the remaining groups (Fig. 5E). The surrounding areas of the dead cells (tumor) corresponding to non-tumor tissue did not show expression of the proapoptotic marker suggesting that the apoptotic machinery is activated locally, at the lesion site, by E_V_DR_L_ (Fig. 5E lower panel).

Taken together, these results show the therapeutic efficacy of E_V_DR_L_ in suppressing tumor growth and prolonging overall survival in mouse models of NSCLC, and that MSCs are a viable therapeutic delivery vehicle for this histological sub-type of lung cancer.

### MSC-E_V_DR_L_ Efficiently Inhibits Leptomeningeal Metastasis Formation in NSCLC-BM Mouse Models

**Figure 6.**
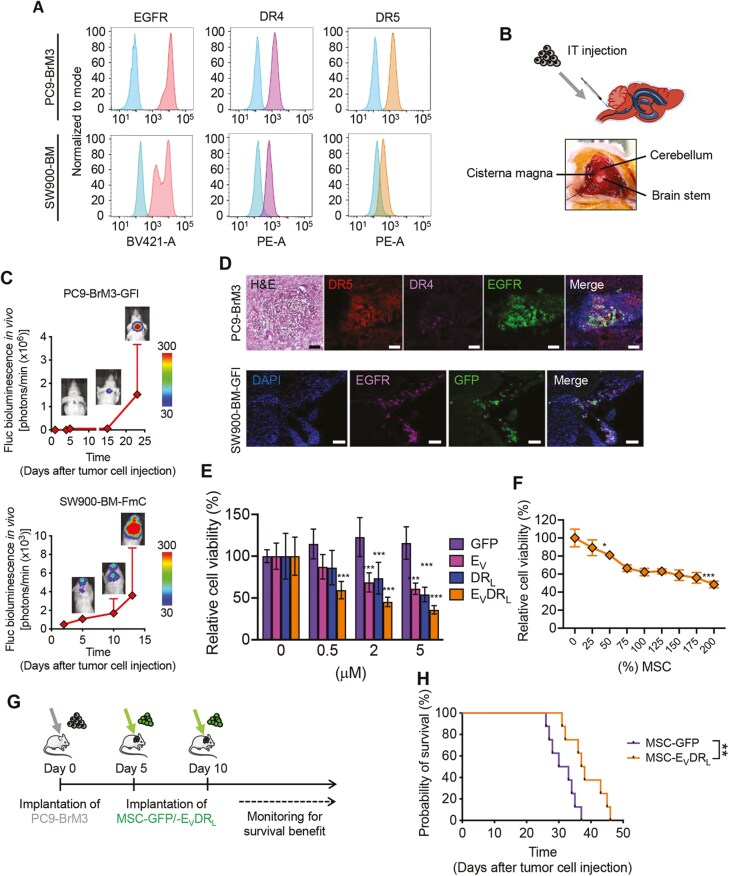
MSCs expressing E_V_DR_L_ efficiently inhibit leptomeningeal formation in NSCLC-BM mouse models. **(A)** FACS analysis of the expression of cell surface markers EGFR, DR4, and DR5 in PC9 BrM3-GFP-Fluc-TK and SW 900 BM-Fluc-mCherry cell lines. Histograms correspond to the medium fluorescence intensity of mAb staining normalized to mode. Analyses conducted twice in duplicate (*n* = 4). **(B)** Schematic of leptomeningeal model in mice that had intra thecal (IT) injection of PC9 BrM3-GFP-Fluc-TK and SW 900 BM-Fluc-mCherry **(C)** BLI signal increase over time and photographs of IT-injected PC9 BrM3-GFP-Fluc-TK (left) and SW 900 BM-Fluc-mCherry (right)-bearing mice **(D)** Representative microphotographs of H&E and immunofluorescence staining with anti-EGFR, anti-DR4 and anti-DR5 antibodies in mice brain of PC9 BrM3-GFP-Fluc cell line (upper panel) and of immunofluorescence staining with anti-GFP and anti-EGFR antibodies in mice brain of SW 900-GFP-Fluc cell line (lower panel). DAPI was used for nuclei fluorescence staining. Pictures show representative fields at 200× magnification. Scale bar = 100 μm. **(E)** PC9 cell line was treated with different concentrations (0, 0.5, 2, and 5 μM) of GFP, E_V_, DR_L_, or E_V_DR_L_, for 72 h and cell viability was determined. Means were calculated from 5 technical replicates. Error bars represent ±S.D. **P < .001 relative to the control GFP. **(F)** Assessment of viability through analysis of FLuc intensity of PC9 BrM3-GFP-Fluc cell line cocultured with increasing percentages (0%-200%, as indicated on the x-axis) of hMSC-E_V_DR_L_. Means were calculated from 3 independent experiments conducted in triplicate (*n* = 9). Error bars represent ±S.D. *P < .05; ***P < .001 relative to tumor cells only used as a control. **(G)** Schematic of the in vivo therapeutic assessment experimental design. Mice were IT-injected with PC9 BrM3-Fluc and treatment with hMSC-GFP or hMSC-E_v_DR_L_ was performed twice specifically 5 and 10 days after tumoral inoculation. **(H)** Kaplan-Meier curves of overall survival of mice bearing PC9 BrM3-GFP-Fluc brain tumors treated with intratumoral injection of hMSC-GFP or hMSC-E_v_DR_L_ (**P < .01).

We tested the therapeutic effect of stem cells secreting E_V_DR_L_ in leptomeningeal metastasis from adenocarcinoma lung tumors. Initially, we confirmed that both brain metastatic PC9 BrM3-GFl and SW 900 BM-FmC cell lines express EGFR, DR4, and DR5 at the cell surface (Fig. 6A). Next, to develop leptomeningeal metastasis in vivo mouse models, we injected these cell lines via intrathecal delivery in the cisterna magna (Fig. 6B). BLI signal showed the successful development of tumors in the brain over time (Fig. 6C). Brains from the mice IT-injected with PC9 BrM3-GFl cell line were then harvested and tested for the upregulation of EGFR, DR4, and DR5 in the tumor area. H&E staining confirmed the presence of brain tumors and immunofluorescence staining with anti-EGFR, anti-DR4, and anti-DR5 antibodies confirmed the expression of the receptors specifically in the neoplastic space (Fig. 6D—upper panel). In addition, anti-EGFR antibodies in mice brains inoculated with SW900-BM-GFl cell line confirmed the increased expression of EGFR in the zone of the tumor formation (Fig. 6D—lower panel). Regarding the therapeutic efficacy of E_V_DR_L_ in NSCLC-BM, PC9-BrM3 cell line was initially tested in vitro for cell viability. After 72 h under E_V_DR_L_ treatment, a significant decrease in the viability of this cell line was observed (Fig. 6E). The same result was obtained with MSC-E_V_DR_L_ cocultured at different ratios with PC9-BrM3 (Fig. 6F). Since implantation of stem cells followed by prolonged survival can increase their ability of tumor formation, we engineered MSC to co-express E_V_DR_L_ and the herpes simplex virus thymidine kinase suicide gene system (HSV-TK). We confirmed that MSC- E_V_DR_L_– TK were eliminated after ganciclovir (GCV) treatment compared with control (Supplementary Fig. S11). The ability of MSC-E_V_DR_L_– TK cells to induce bystander effect in NSCLC-BM was tested by coculture with SW900-BM-GFP-Fluc cells. A pronounced decrease in the total cell viability was observed after GCV treatment depicting an increased tumor-killing effect by MSC-E_V_DR_L_– TK cells, and an enhanced therapeutic benefit of TK in these cells (Supplementary Fig. S12). In vivo, treatment with MSC-E_V_DR_L_ resulted in significant improvement in survival of PC9 BrM3 tumor-bearing mice (Fig. 6G, 6H). Overall, we demonstrated the therapeutic efficacy of IT-delivered MSC- E_V_DR_L_ in mouse models of NSCLC-LM.

## Discussion

In this study, we tested simultaneous stimulation of death receptor-mediated apoptosis and inhibition of EGFR downstream signaling via MSC-mediated release of the bi-functional molecule E_V_DR_L_ as a new strategy for the treatment of NSCLC and LM resulting from NSCLC. Both EGFR and DR4/5 receptors have been shown to be overexpressed in NSCLC samples. We show that E_V_DR_L_ efficiently inhibits the activation of signal transduction cascades downstream of EGFR while also activating caspase-mediated apoptosis in the vast majority of NSCLC cell lines we tested. Furthermore, we show that intravenously injected MSC-E_V_DR_L_ home to NSCLC tumors in the lung lobules and metastatic sites in the brain of mice, significantly reduce tumor burden and extend survival. Currently, the approved treatment of EGFR-positive NSCLC patients consists of 3 generations of EGFR-TKIs. The most common *EGFR* mutations, exon 19 deletion (E746-A750), and the L858R point mutation (exon 21) represent the main targets for first- and second-generation EGFR TKIs and are established standard-of-care therapies for patients harboring such mutations. However, development of resistance to EGFR TKIs, such as an acquisition of “gatekeeper” mutation *EGFR* T790M, is a frequent feature of disease progression.^6-8,42,43^ Moreover, some patients eventually progress even on 3rd-generation covalent TKIs such as osimertinib, via acquisition of the C797S mutation.^44-46^ Our findings indicate that E_V_DR_L_ therapy was highly effective in the treatment of mice bearing xenografts from wild-type EGFR cell lines. Also, it prominently induces cell death in double-mutant EGFR T790M/L858R H1975 cell line, as well as PC9-Bm3 cell line characterized by a Glu746-Ala750 deletion on exon 19 (Supplementary Fig. S13).^47^ Importantly, we did not test the therapeutic efficacy of E_V_DR_L_ therapy in mice bearing xenographs from EGFR mutant cell lines and future studies will address this point. However, taking into consideration the ongoing efforts to overcome EGFR resistance mechanisms,^44^ a dual-targeted therapy like E_V_DR_L_ represents a promising strategy as it can block both wild-type and mutant EGFR in vitro, and likely would not lead to the common resistance mechanisms seen with TKIs.

Activation of intracellular EGFR signaling components such as PI3K-AKT or ERK MAPK promotes cell proliferation and induction of resistance to apoptotic events, as we and others have previously shown.^48-52^ For example, ERK negatively regulates apoptosis by phosphorylation of the pro-apoptotic protein BIM on S69, as well as AKT’s anti-apoptotic role by direct phosphorylation of proteins of the cell death machinery (such as BAD).^53^ We measured the signal transduction pathways downstream of EGFR and DR4/5 and found that increasing concentrations of E_V_DR_L_ resulted in decreased expression of phosphorylated EGFR and to a lesser extent AKT and ERK; however, E_V_ alone did not have any efficacy in reducing proliferation of EGFR-driven NSCLC cell lines. These findings collectively suggest that the primary role of the E_V_ domain of E_V_DR_L_ might be enhancing DR_L_’s apoptosis-inducing effect rather than blocking EGFR signaling. Furthermore, replacement of anti-EGFR nanobody by anti-EGFR single-chain variable fragment (E_S_DR_L_) antibody domain did not result in therapeutic benefit compared with E_V_DR_L_ treatment, suggesting that the specific combination of targeting cell proliferation and death pathways in tumor cells is essential. DR_L_ selectively targets and induces apoptosis in tumor cells but not in most healthy, non-neoplastic cells.^12,54^ By binding to its receptors DR4 and DR5, DR_L_ activates a caspase-8-mediated apoptotic cascade causing tumor cell death.^55^ In fact, caspase-8 as an initiator caspase cleaves and activates the effector caspases-3, -6, and -7 located downstream of the cascade, which results in the proteolytic disassembly of cells.^12,56^ We observed the cleavage of both initiator and effector caspases after treatment with E_V_DR_L_, suggesting that the intrinsic pathway is involved in E_V_DR_L_-induced apoptosis. Moreover, E_V_DR_L_ exerted its apoptotic effect in a time-dependent manner for different NSCLC lines. For the SW900 cell line, the majority of caspases were activated after an 8-hour treatment, whereas in H2170 cells apoptosis mainly occurred after 24 h.

Our in vivo studies showed that intravenously injected MSCs engineered to secrete E_V_DR_L_ migrate to NSCLC tumors and significantly reduce tumor burden. We found that in ­non-tumor-bearing animals, MSC engraftment was very low, and their bioluminescent signal disappeared on average 24 h after post-I.V. implantation. In the majority of tumor-bearing animals, MSCs could still be detected 96 h after inoculation but not after 144 h, which could perhaps be extended by delivery of a larger number of cells and/or multiple injections over the course of an established timeframe. It has been widely demonstrated by us and other laboratories that MSCs home to tumors in several models such as breast cancer and its BM,^35^ gliomas,^57^ lung metastases,^18,34^ melanoma and its BM,^58^ Kaposi sarcomas,^59^ and xenograft mouse models of lung cancer.^60^ Although the exact mechanism behind this innate tropism is still not fully understood, reports from in vitro and in vivo mouse models suggest that tumors release certain chemokines recognized by receptors displayed on the MSC cell surface.^60-63^

MSCs administered intravenously are primarily trapped in the lungs. We and others observed the redistribution of MSCs from the lungs to the liver as soon as 24 h after administration.^64^ Although MSCs are cleared relatively quickly even in the presence of tumors, this time frame seems to be enough to elicit a therapeutic benefit. In immunocompetent pre-clinical mouse models, it has been suggested that MSCs rapidly impact resident cells which subsequently prompt an immunomodulatory and beneficial response. We took advantage of the homing capability of MSCs to deliver E_V_DR_L_ into the tumor microenvironment, providing survival benefits in both primary and LM-NSCLC mouse models. These results were encouraging and supported by the early separation of the Kaplan-Meier curves of overall survival between E_V_DR_L_ and control (GFP) therapies.

Current treatment for NSCLC typically involves surgical resection together with neoadjuvant and/or adjuvant therapy, including targeted therapy. The phase III FLAURA clinical trial showed that the treatment of patients with advanced stage NSCLC harboring EGFR mutations with next-generation TKI (such as osimertinib) versus standard EGFR-TKI resulted in significantly longer progression-free survival (PFS).^45^ In another randomized phase III clinical trial, the beneficial effects of combining recombinant soluble human DR_L_ concurrently with vinorelbine and cisplatin (chemotherapeutic cocktail) in untreated patients with stage IIIB/IV advanced or recurrent NSCLC show improved PFS and overall response rate (ORR) but not overall survival.^65^ Notably, the DR4/5 agonist dulanermin displays a short half-life and rapid clearance when administered systemically, which could have contributed to the poor survival outcome in this study.^65^

We found IT-delivered stem cell therapy to be a promising approach for treating LM from NSCLC, a secondary neoplasia of the CNS currently having as primary treatment palliative care.^3,66^ IT-injected stem cells secreting E_V_DR_L_ into cerebrospinal fluid (CSF), provided survival benefits in our mouse model of lung cancer-associated LM without affecting the general health of the mouse. IT-delivered stem cell therapy has been considered as a safe and well-tolerated approach in the treatment of patients with multiple system atrophy (MSA),^67^ multiple sclerosis,^68^ amyotrophic lateral sclerosis,^69^ stroke,^70^ epilepsy.^71^ In addition, IT administration of MSCs showed therapeutic benefit in mouse models with leptomeningeal dissemination from gliomas,^72^ and medulloblastomas.^73^

MSCs as therapeutic delivery vectors have become increasingly popular due to their attractive characteristics: they can be easily extracted from bone marrow and transduced with viral vectors without altering their stemness properties,^74^ and they display low immunogenicity as they lack the expression of MHC II or co-stimulatory molecules CD80, CD86, and CD40.^75^ In 2 separate in vivo studies, MSCs expressing DR_L_ provided a significant reduction of tumor burden in metastatic and primary xenograft lung tumor mouse models.^18,60^ A recent phase I/II clinical trial, TACTICAL, is evaluating the anti-tumor activity of MSC-expressing Trail in addition to pemetrexed/cisplatin chemotherapy in metastatic NSCLC patients (NCT03298763, clinicaltrials.gov). Despite the clear advantages of SC-based therapies, there are challenges toward clinical translation.^76^ Primarily, MSCs have reduced survival following in vivo transplantation, limiting treatment efficacy.^77^ To overcome this, autologous SC transplantation could prevent premature clearance by the recipient’s immune system.

In conclusion, we demonstrated that MSC-mediated delivery of E_V_DR_L_ represents a viable therapeutic strategy for the treatment of NSCLC and NSCLC-LM. We envision that this therapy could be developed in the adjuvant setting following surgical resection of the primary neoplasia, and future studies should analyze how this approach influences tumor behavior and its microenvironment to better mimic this proposed clinical application.

## Supplementary Material

szad033_suppl_Supplementary_Figures

szad033_suppl_Supplementary_Material

## Data Availability

The processed data are provided in the Figures. Additional data requests can be made to the corresponding author.
