# Expression of Concern: Fate and Efficacy of Engineered Allogeneic Stem Cells Targeting Cell Death and Proliferation Pathways in Primary and Brain Metastatic Lung Cancer

**DOI:** 10.1093/stcltm/szae012

**Published:** 2024-02-05

This is an Expression of Concern regarding:

Susana Moleirinho, Yohei Kitamura, Paulo S G N Borges, Sophia Auduong, Seyda Kilic, David Deng, Nobuhiko Kanaya, David Kozono, Jing Zhou, Jeffrey J Gray, Esther Revai-Lechtich, Yanni Zhu, Khalid Shah, Fate and Efficacy of Engineered Allogeneic Stem Cells Targeting Cell Death and Proliferation Pathways in Primary and Brain Metastatic Lung Cancer, *Stem Cells Translational Medicine*, Volume 12, Issue 7, July 2023, Pages 444–458, https://doi.org/10.1093/stcltm/szad033

The Journal has been made aware of concerns raised by a reader regarding images in Figure 2B of this paper. The Journal is publishing this expression of concern while an investigation is carried out.

