# Correction to: Fate and Efficacy of Engineered Allogeneic Stem Cells Targeting Cell Death and Proliferation Pathways in Primary and Brain Metastatic Lung Cancer

**DOI:** 10.1093/stcltm/szaf021

**Published:** 2025-05-26

This is a correction to: Susana Moleirinho, Yohei Kitamura, Paulo S G N Borges, Sophia Auduong, Seyda Kilic, David Deng, Nobuhiko Kanaya, David Kozono, Jing Zhou, Jeffrey J Gray, Esther Revai-Lechtich, Yanni Zhu, Khalid Shah, Fate and Efficacy of Engineered Allogeneic Stem Cells Targeting Cell Death and Proliferation Pathways in Primary and Brain Metastatic Lung Cancer, *Stem Cells Translational Medicine*, Volume 12, Issue 7, July 2023, Pages 444–458, https://doi.org/10.1093/stcltm/szad033

In February 2024 the Journal published an Expression of Concern^1^ regarding concerns raised about Figure 2B via PubPeer (https://pubpeer.com/publications/693E59E7A3C195220101CCAAE695E2). Shortly thereafter, concerns were also raised about Figure 3B via PubPeer (https://pubpeer.com/publications/693E59E7A3C195220101CCAAE695E2#2).

As part of the inquiry into Figure 2B, the authors retrieved the cell culture images for the SW900 and H27170 lines and explained that duplication occurred whilst compiling the images. A revised version of Figure 2B from the authors is provided below.


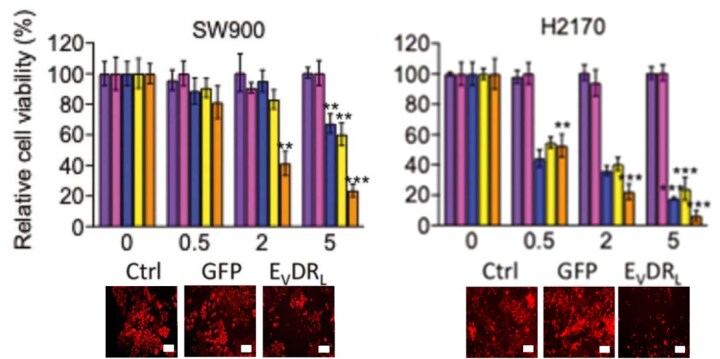


As a part of the inquiry into Figure 3B, the authors explained they performed multiple blots for each experiment and made an error in matching the blot shown in Figure 3B to the raw blots shown in the H2170 line in Supplementary Figure 14. A revised version of Figure 3B reflecting the revised H2170 panel, as well as a revised version of Supplementary Figure 14 showing updated raw western blots, from the authors are provided below.


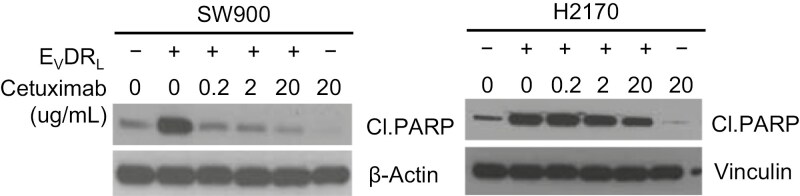



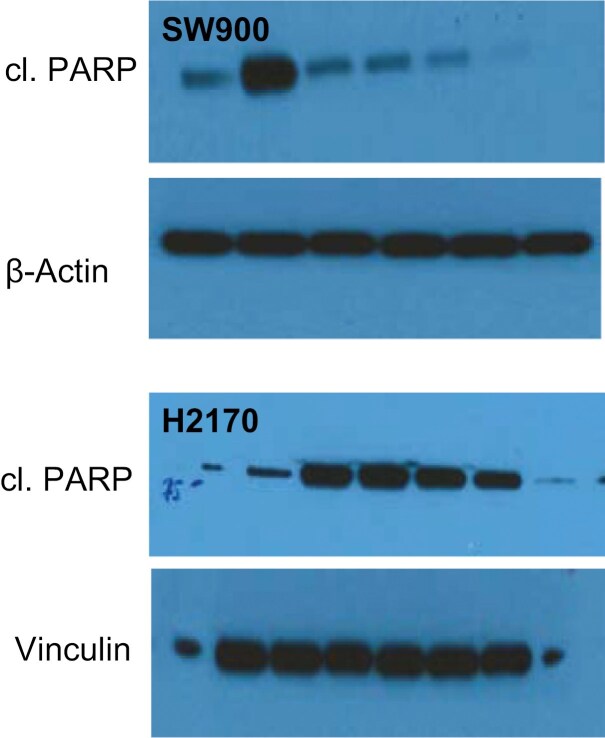


The Editors agree with the authors’ assertions that the findings of the paper are unaffected by these errors. The correction has been made only in this correction notice so as to preserve the published Version of Record.

^1^ Expression of Concern: Fate and Efficacy of Engineered Allogeneic Stem Cells Targeting Cell Death and Proliferation Pathways in Primary and Brain Metastatic Lung Cancer, *Stem Cells Translational Medicine*, 2024, szae012, https://doi.org/10.1093/stcltm/szae012

