# Supplementary material for: Fate and Efficacy of Engineered Allogeneic Stem Cells Targeting Cell Death and Proliferation Pathways in Primary and Brain Metastatic Lung Cancer
Source: Stem Cells Transl Med. 2023 Jun 13;12(7):444–58. doi: 10.1093/stcltm/szad033 (PMC10346421; doi:10.1093/stcltm/szad033)
Supplement: szad033_suppl_Supplementary_Figures [file szad033_suppl_supplementary_figures.pdf]

Supplementary Fig.1

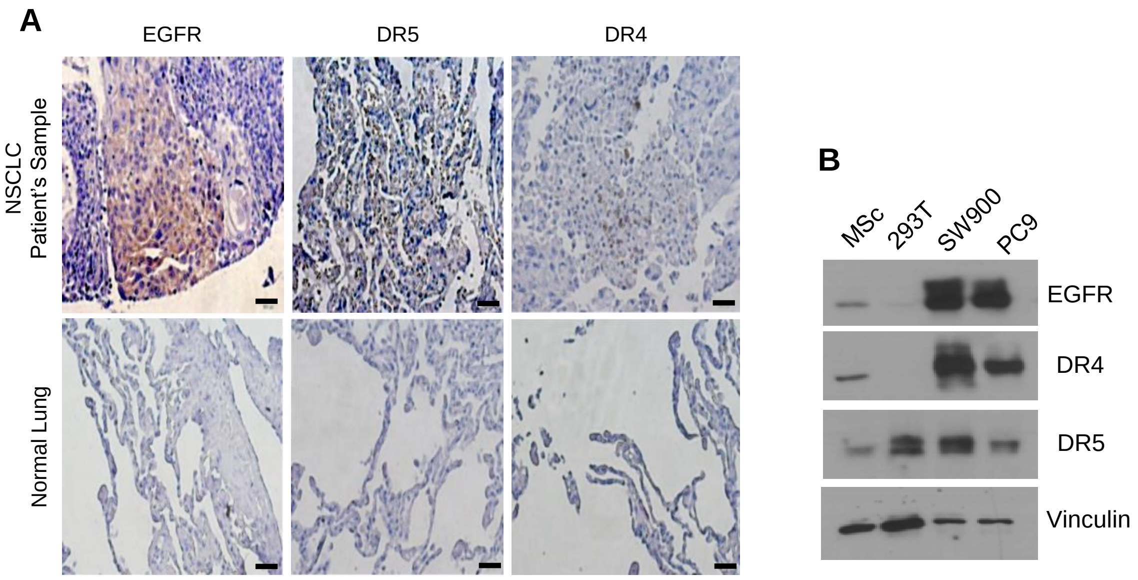

Figure S1. Related to Figure 1. A. Immunohistochemistry images of NSCLC patient tissue vs healthy samples stained with anti-EGFR, anti-DR4, and anti-DR5 antibodies. Pictures show representative fields at 200x magnification. Scale bar = 200μm. **B.** Western blotting showing the expression of EGFR, DR4 and DR5 in normal and lung tumor cells.

Supplementary Fig. 2

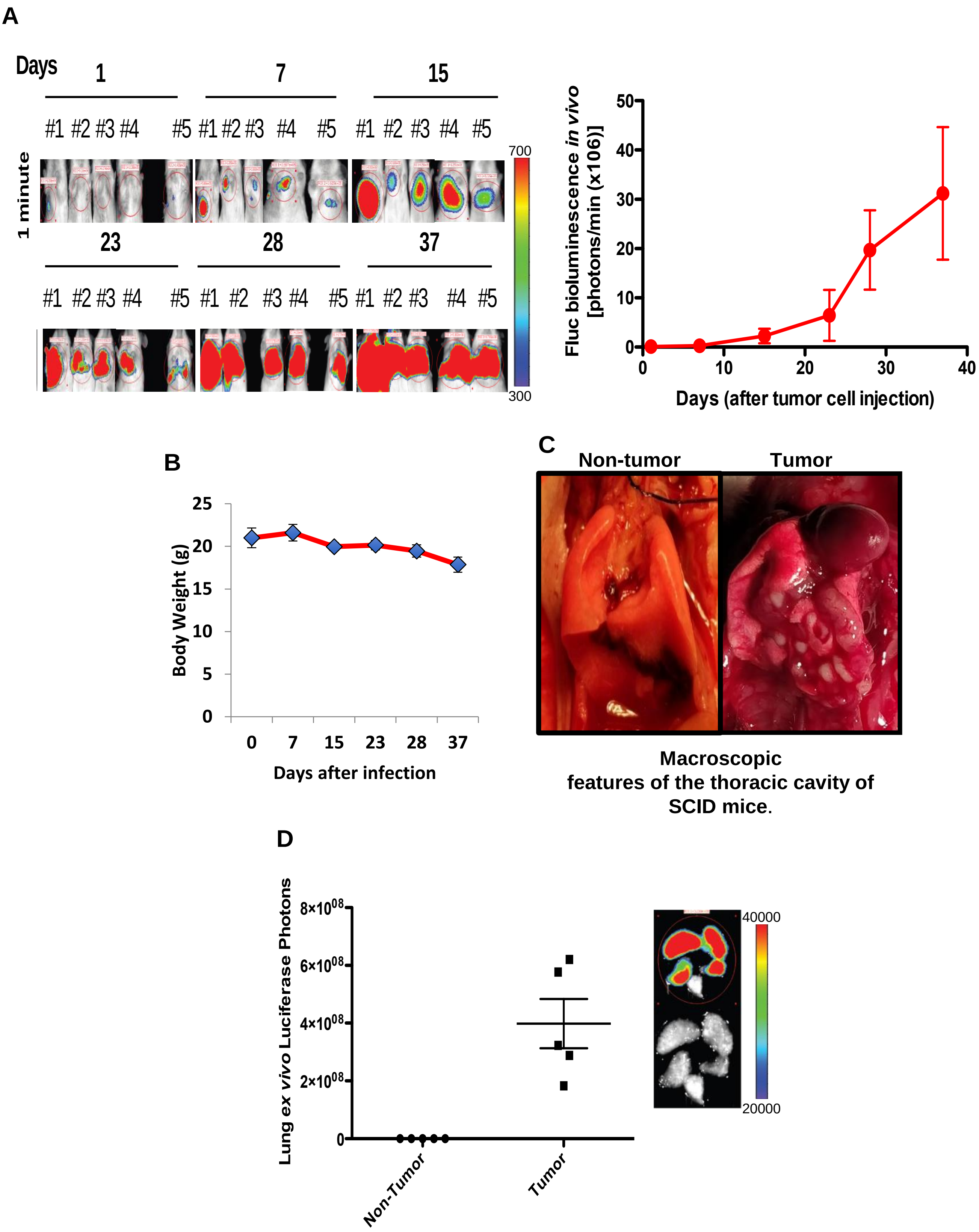

Figure S2. Related to Figure 1. Characterization of NSCLC mouse tumor model (A) *In vivo* plot of bioluminescence imaging showing tumor growth and its quantification in immunocompromised NOD/SCID or athymic nude mice bearing SW900-FmC (0.5 x 10^6 cells) (*n* = 5 animals). **(B)** Body weights of mice inoculated with SW900-FmC (displayed in A) over time. **(C)** Macroscopic features of the thoracic cavity in tumor or non-tumor-bearing NOD-SCID mice (*n* = 2 animals each group). **(D)** *Ex vivo* analysis. Tumor or non-tumor-bearing lungs were removed and subjected to luciferin treatment for about 10 minutes before BLI (*n* = 5 animals each group).

Supplementary Fig. 3

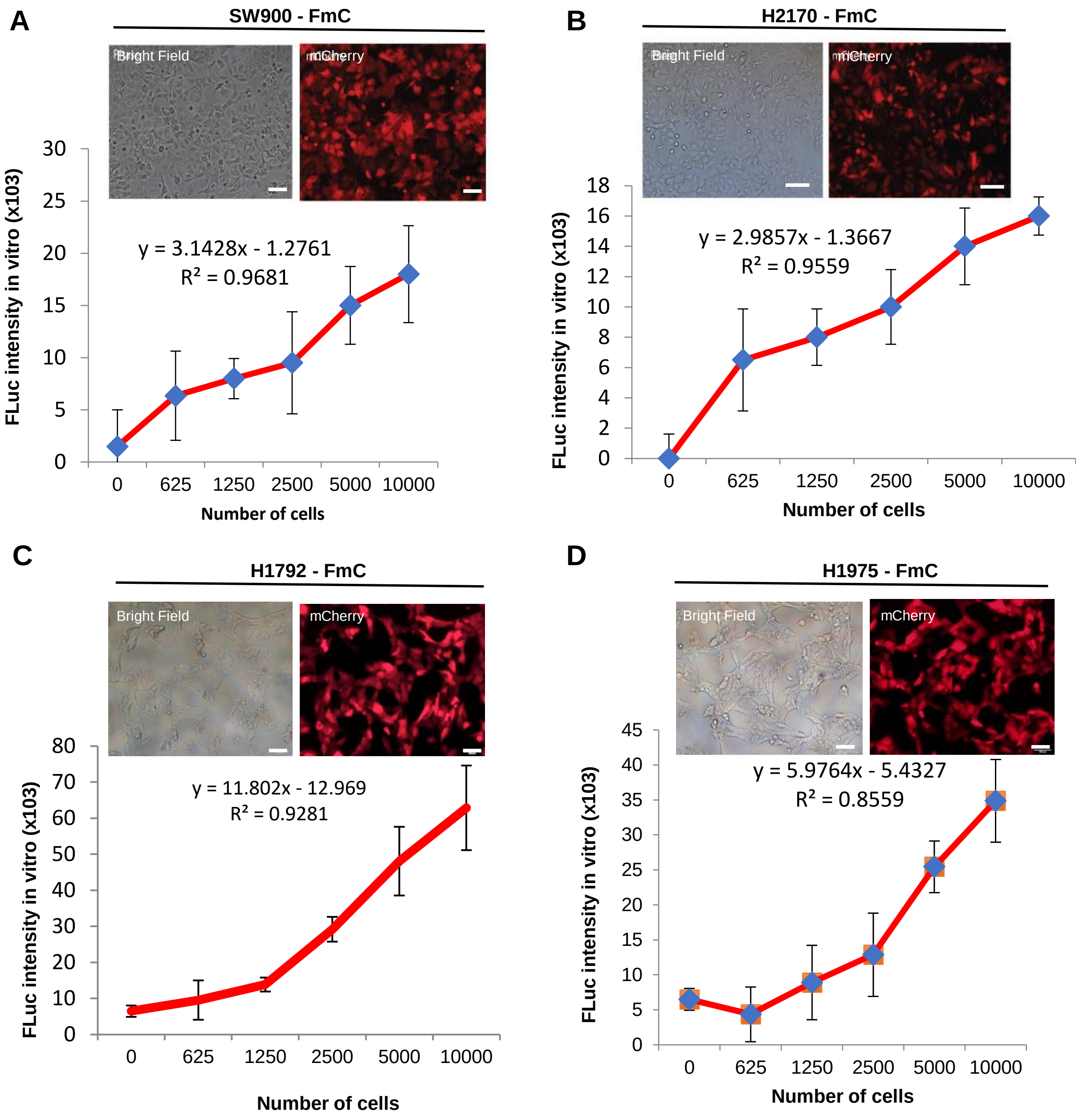

Figure S3. Related to Figure 2, Figure 4, and Figure 5. Engineering of cell lines for *in vivo* and *ex vivo* BLI and *in vitro* downstream analyses. (A-D) SW900, H2170, H1792, and H1975 cell lines were engineered with lentiviral Fluc-mCherry (FmC) constructs allowing the simultaneous acquisition of fluorescence and bioluminescence signal. Graphs show the correlation of Fluc signal intensity with number of cells plated per well. Means were calculated from two independent experiments conducted in triplicate (*n*=6). Error bars represent ±S.D. Scale bars = 100 µm.

Supplementary Fig. 4

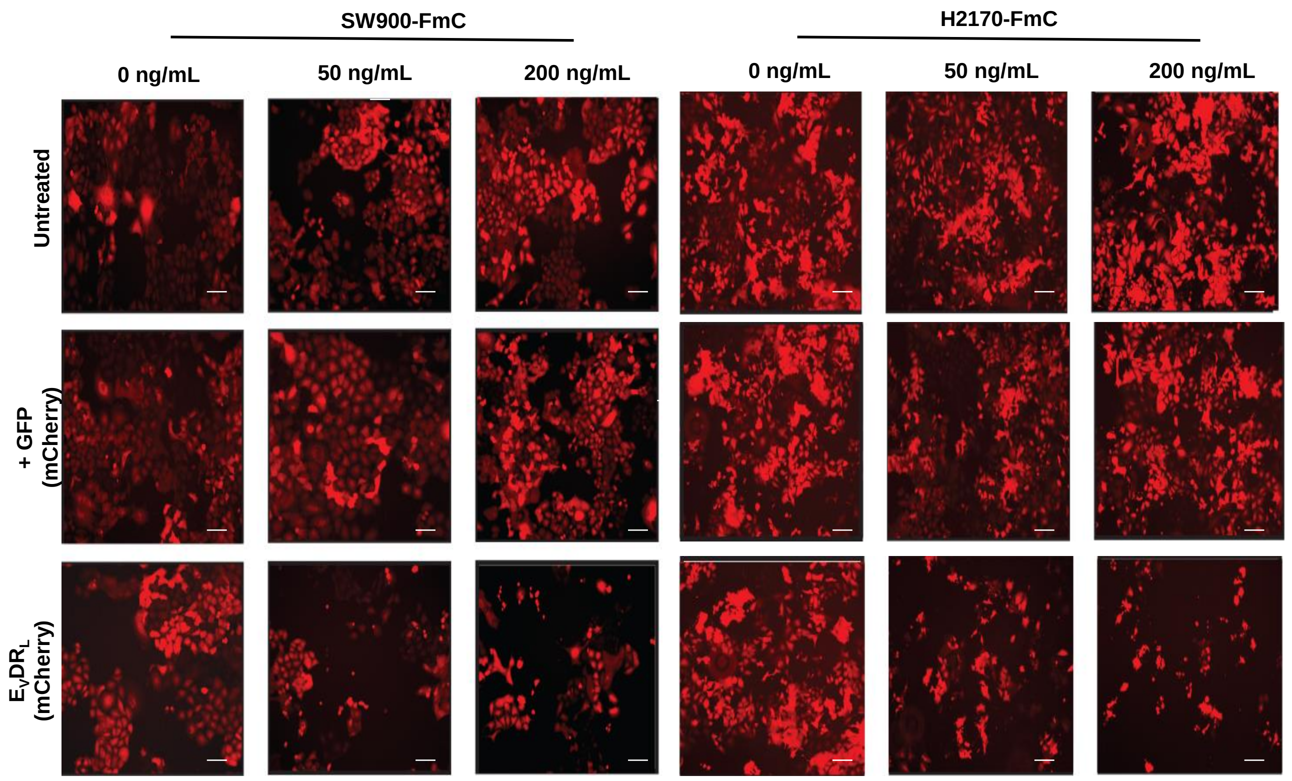

Figure S4. Related to Figure 2. NSCLC cell lines treated with E_VDR_L show significant loss of cell viability. (A) Cell viability was determined after SW900-FmC and H2170-FmC cell lines were treated with different concentrations (0, 0.5, 2, 5 µM) of GFP or E_VDR_L, for 72 h. Phase contrast photomicrographs show prominent loss of cell viability after E_VDR_L treatment compared with control treatments at the same conditions or untreated cell lines. Scale bars = 100 µm.

Supplementary Fig. 5

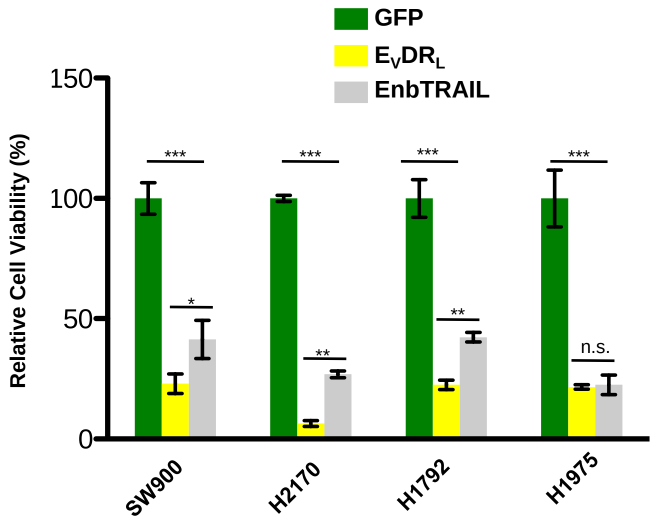

Figure S5. Related to Figure 2. E$_V$DR$_L$ shows higher cytotoxicity to NSCLC cell lines compared to ENbTrail. (A) Schematic representation of lentiviral transfer vectors composed by cytotoxic variant of EGFR nanobody, ENb-Trail containing an Flt3 domain or a cytotoxic extracellular domain of TRAIL (DR$_L$) fused to a linker sequence and a leucine zipper domain only. **(B)** SW900, H2170, H1792, and H1975 cell lines cells were treated with 5 μM of GFP, ENb-TRAIL or E$_V$DR$_L$ for 72 h and cell viability was determined. Means were calculated from two independent experiments conducted in triplicate (*n*=6). Error bars represent ±S.D. * $p < 0.05$; ** $p < 0.01$; *** $p < 0.001$; n.s. – non-significant relative to the control GFP.

Supplementary Fig. 6

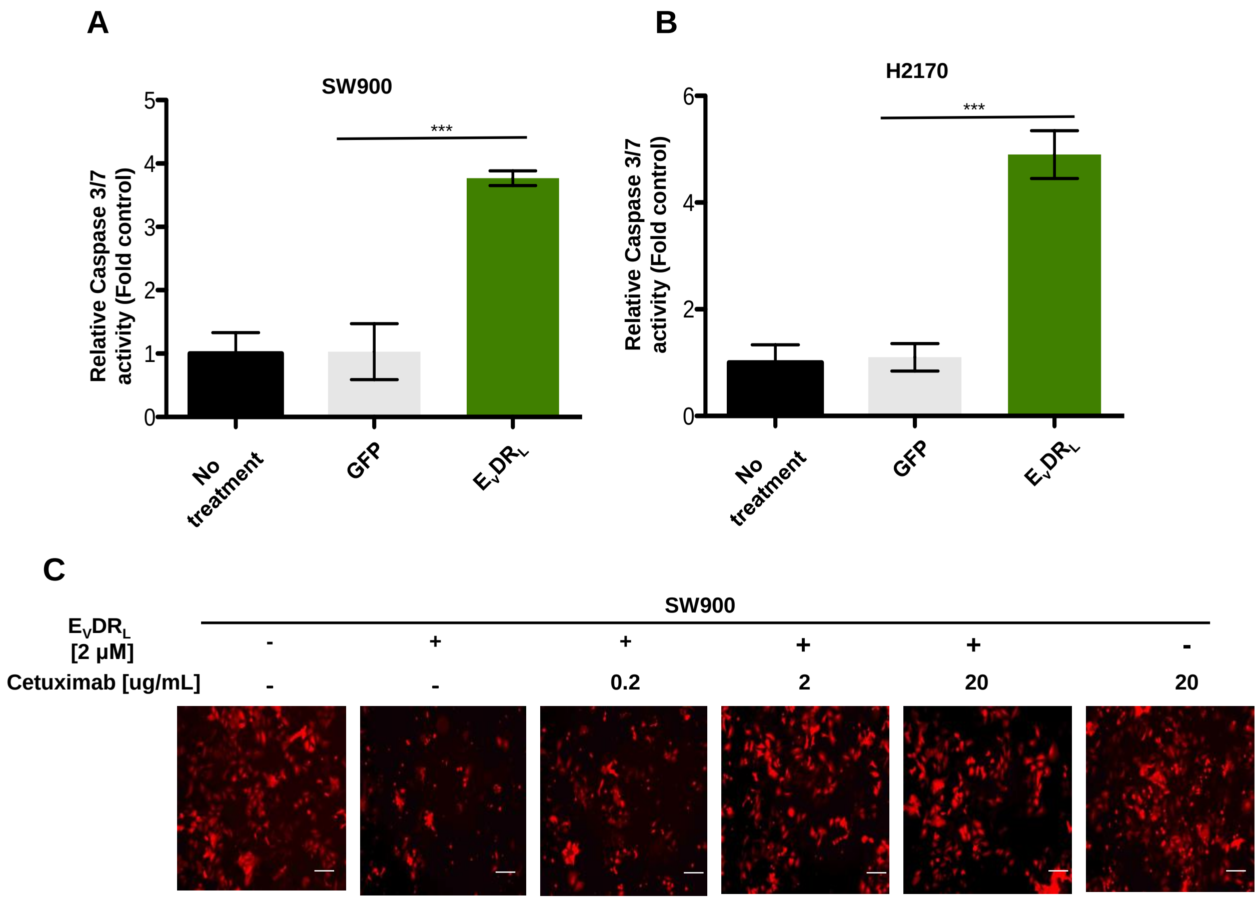

Figure S6. Related to Figure 3. (A-B) Relative caspase 3/7 activity analysis on SW900 and H2170 cell lines treated with either GFP or E_VDR_L (2 μM) for 8 hours. Non-treated cells were used as additional control. Means were calculated from three independent experiments conducted in triplicate (*n*=9). Error bars represent ±S.D. *** $p < 0.001$. **(C)** Phase contrast photomicrographs of SW900-FmC cell line incubated with 0, 0.2, 2, or 20 ug/mL of cetuximab for 30 minutes followed by treatment with E_VDR_L for 24h (2 μM). Pictures show representative fields at 100x magnification. Scale bar = 100 μm.

Supplementary Fig. 7

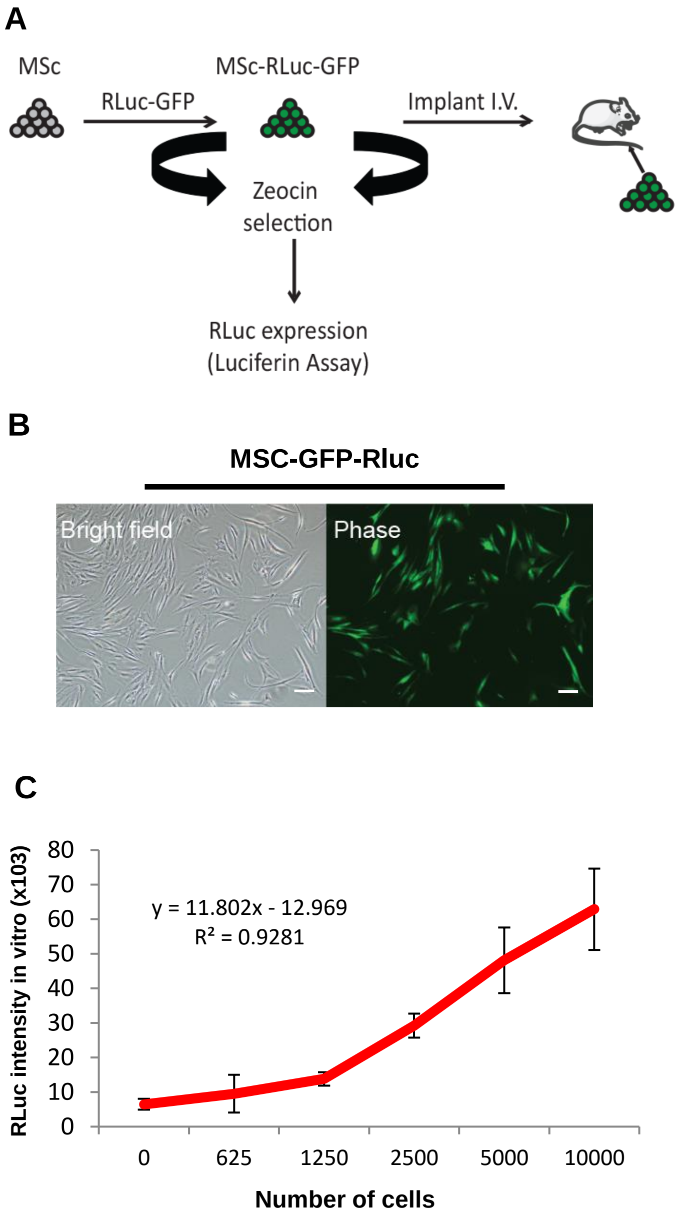

Figure S7. Related to Figure 4. (A) MSCs were engineered with lentiviral Rluc-GFP constructs followed by zeocin selection (100 µg/mL). MSCs stably expressing Rluc-GFP (monoclonal population) were tail I.V. injected in either NOD/SCID or athymic nude mice. **(B)** Bright field and phase contrast photomicrographs (GFP) showing successful transduction of MSCs. Pictures show representative fields at 100x magnification. Scale bar = 50 µm. **(C)** Graph showing the correlation of Rluc signal intensity with number of cells plated per well. Means were calculated from two independent experiments conducted in triplicate (*n*=6). Error bars represent ±S.D.

Supplementary Fig. 8

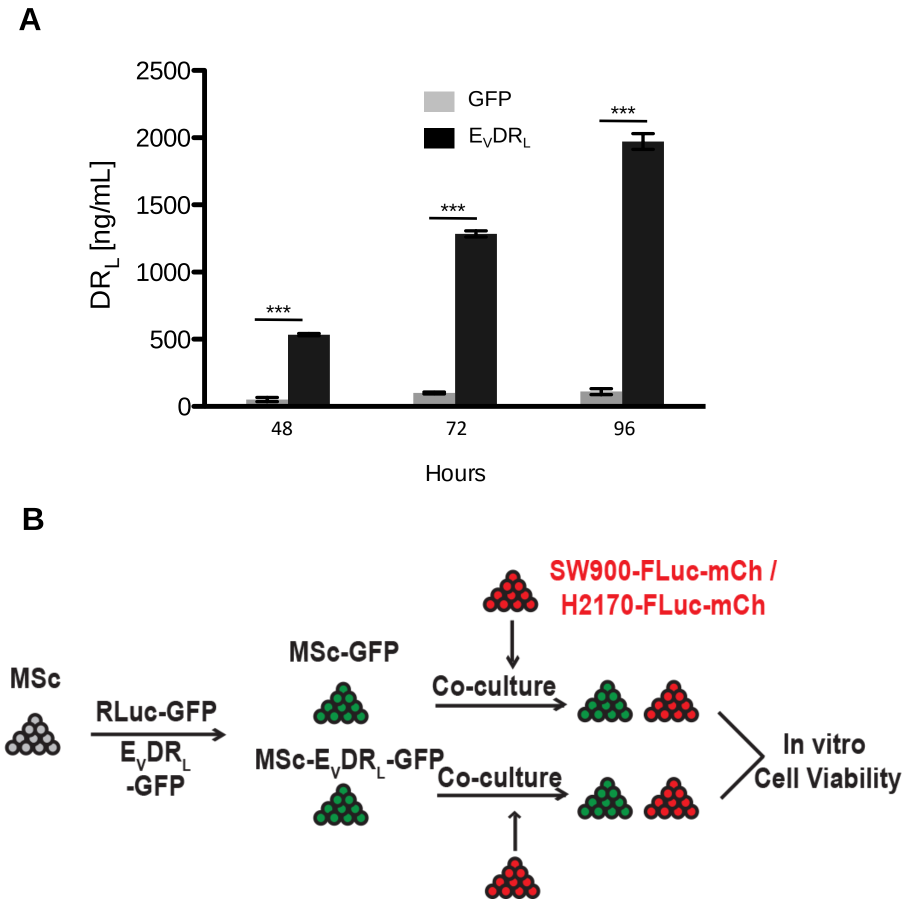

Figure S8. Related to Figure 5. (A) Elisa quantification of secreted DR_L from MSC-expressing E_VDR_L compared with MSC-GFP only cells. Cells were plated and conditioned medium was collected at the indicated time points. Means were calculated from two independent experiments conducted in triplicate (n=6). Error bars represent ±S.D. *** $p < 0.001$. **(B)** Schematic of the co-culture experimental design. MSCs transduced with either E_VDR_L or GFP only were co-cultured with SW900-FmC or H2170-FmC at the indicated ratios relative to the tumor cells for 72h.

Supplementary Fig. 9

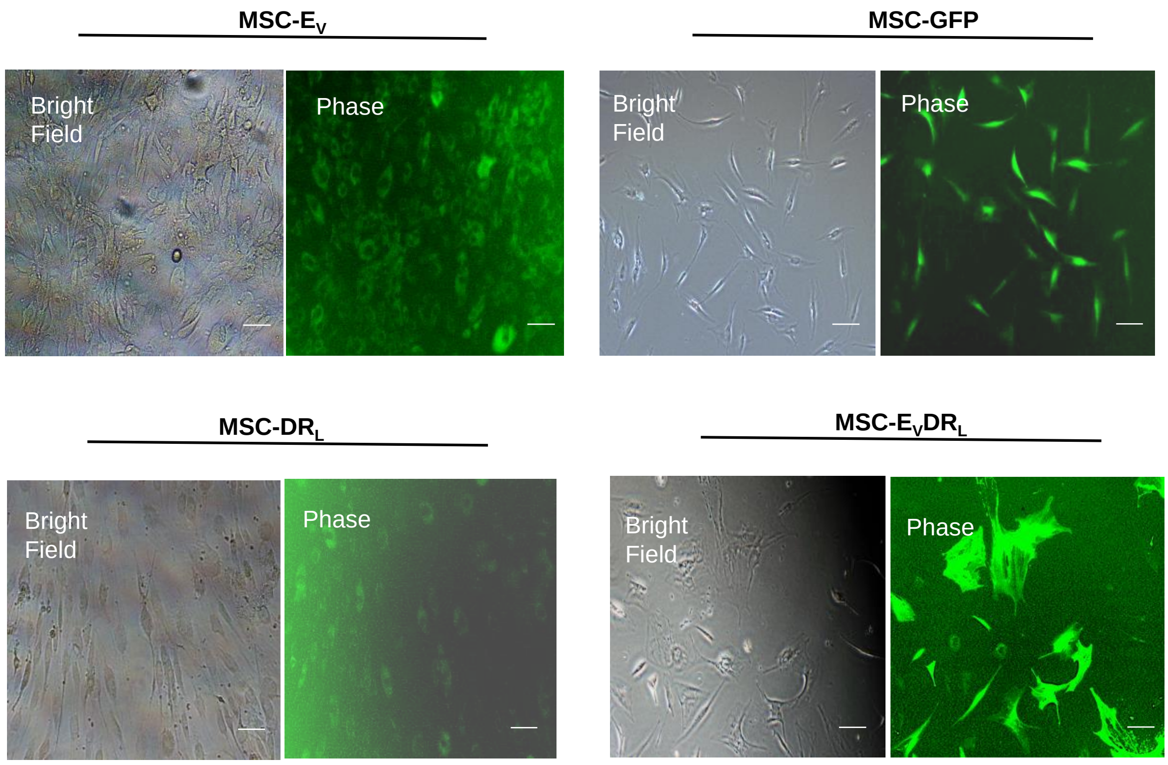

Figure S9. Related to Figure 5. Bright field and phase contrast photomicrographs (GFP) showing successful transduction of MSC with E_v only, GFP only, DR_L or E_vDR_L. Pictures show representative fields at 100x magnification. Scale bar = 100 µm.

Supplementary Fig. 10

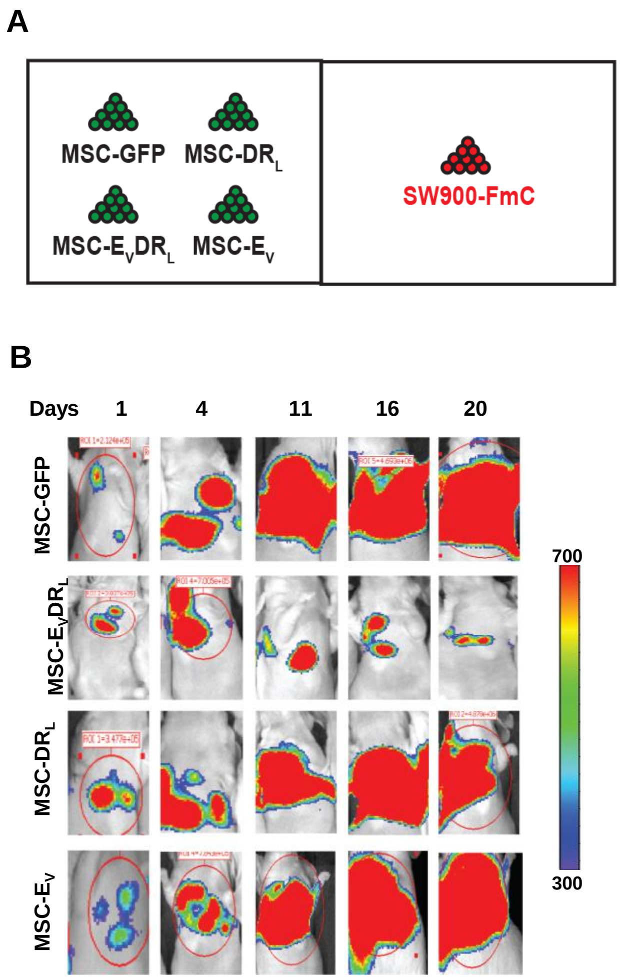

Figure S10. Related to Figure 5. (A) Schematic of the *in vivo* MSCs-expressing GFP; DR$_L$; E$_V$ or E$_V$DR$_L$ cells used in the therapeutic assessment experimental design. **(B)** E$_V$DR$_L$ efficiently suppresses tumor volume in the lung. Representative BLIs of mice tumor volume from each treatment group before and after MSC implantation (pre-treatment – days 1 and 4; post-treatment – days 11, 16, and 20).

Supplementary Fig. 11

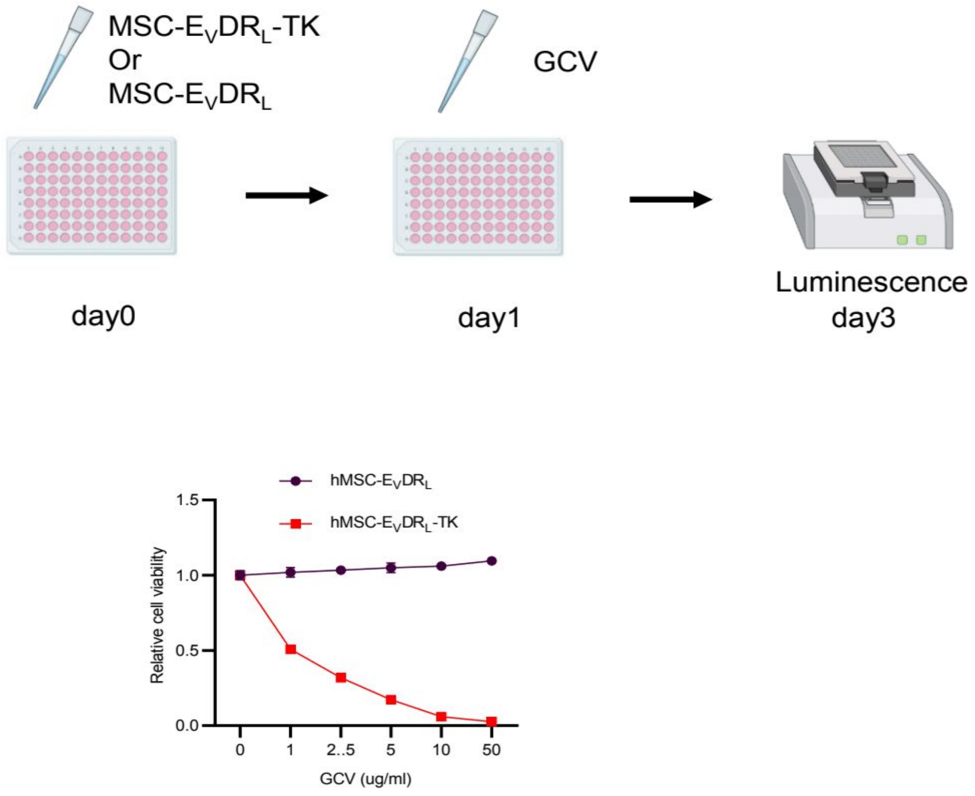

Figure S11. Related to Figure 6. Cell viability assay of MSC-E_VDR_L in the presence or absence of GCV for 48h (n = 5 per group) shows that MSC expressing HSV-TK can be eradicated post GCV treatment.

Supplementary Fig. 12

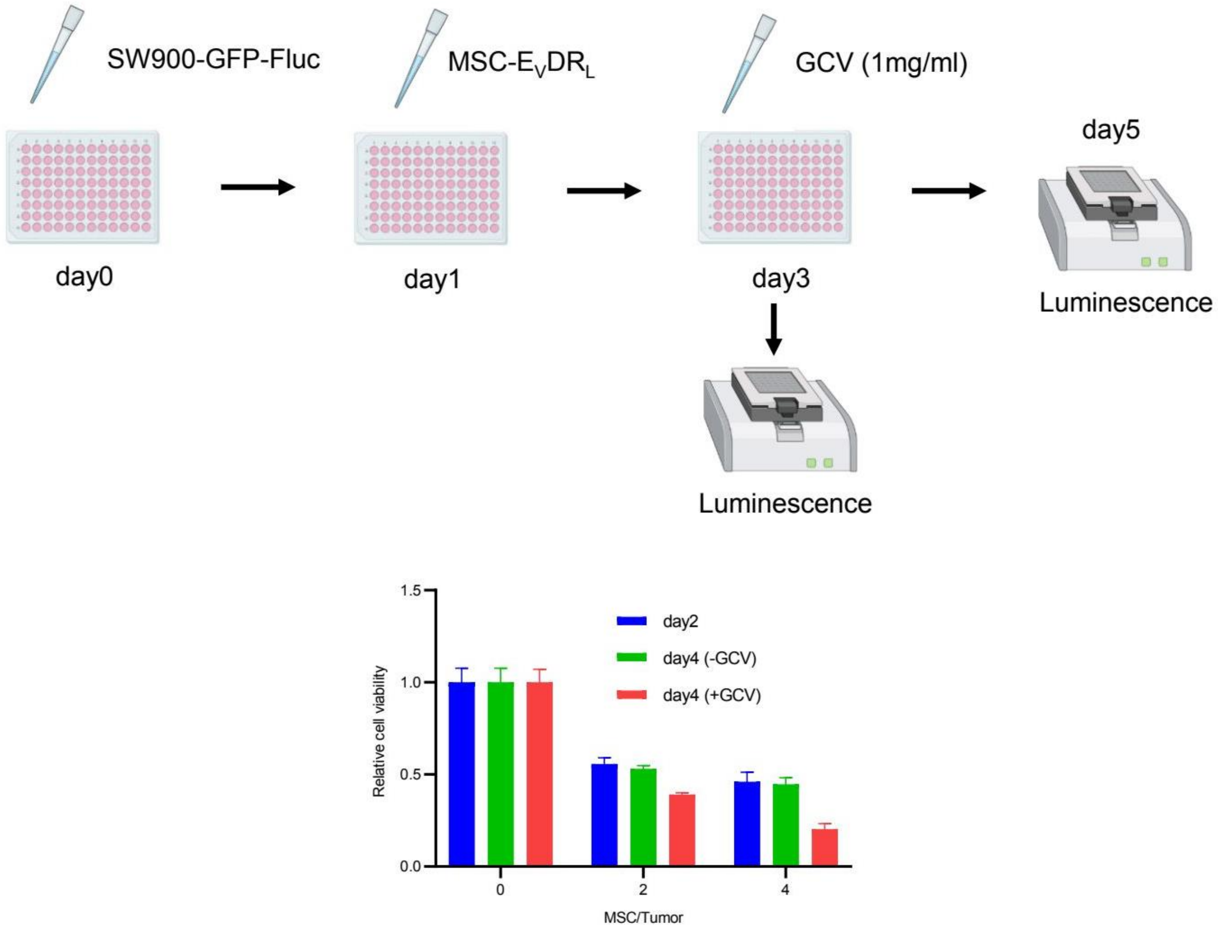

Figure S12. Related to Figure 6. MSC expressing HSV-TK induces bystander effect in lung tumor cells. Cell viability assay of SW900-GFl cells co-cultured with MSC-EVDR-TK for 48h, followed by 48h incubation with GCV (1 mg/ml) (n = 5 per group). *:p<0.001.

Supplementary Fig. 13

Cell Lines	Histology	EGFR Mutation Type
SW900	Squamous carcinoma	Wild type
H2170	Squamous carcinoma	Wild type
H358	Bronchioalveolar carcinoma	Wild type
A549	Adenocarcinoma	Wild type
H23	Adenocarcinoma	Wild type
H1975	Adenocarcinoma	T790M/L858R
H1792	Adenocarcinoma	Wild type
PC9-BrM3	Adenocarcinoma	Glu746-Ala750 deletion

Figure S13. Related to Figure 1, Figure 2, Figure 3, Figure 4, Figure 5, and Figure 6. EGFR mutation status of NSCLC cell lines used in this study.

Supplementary Fig. 14

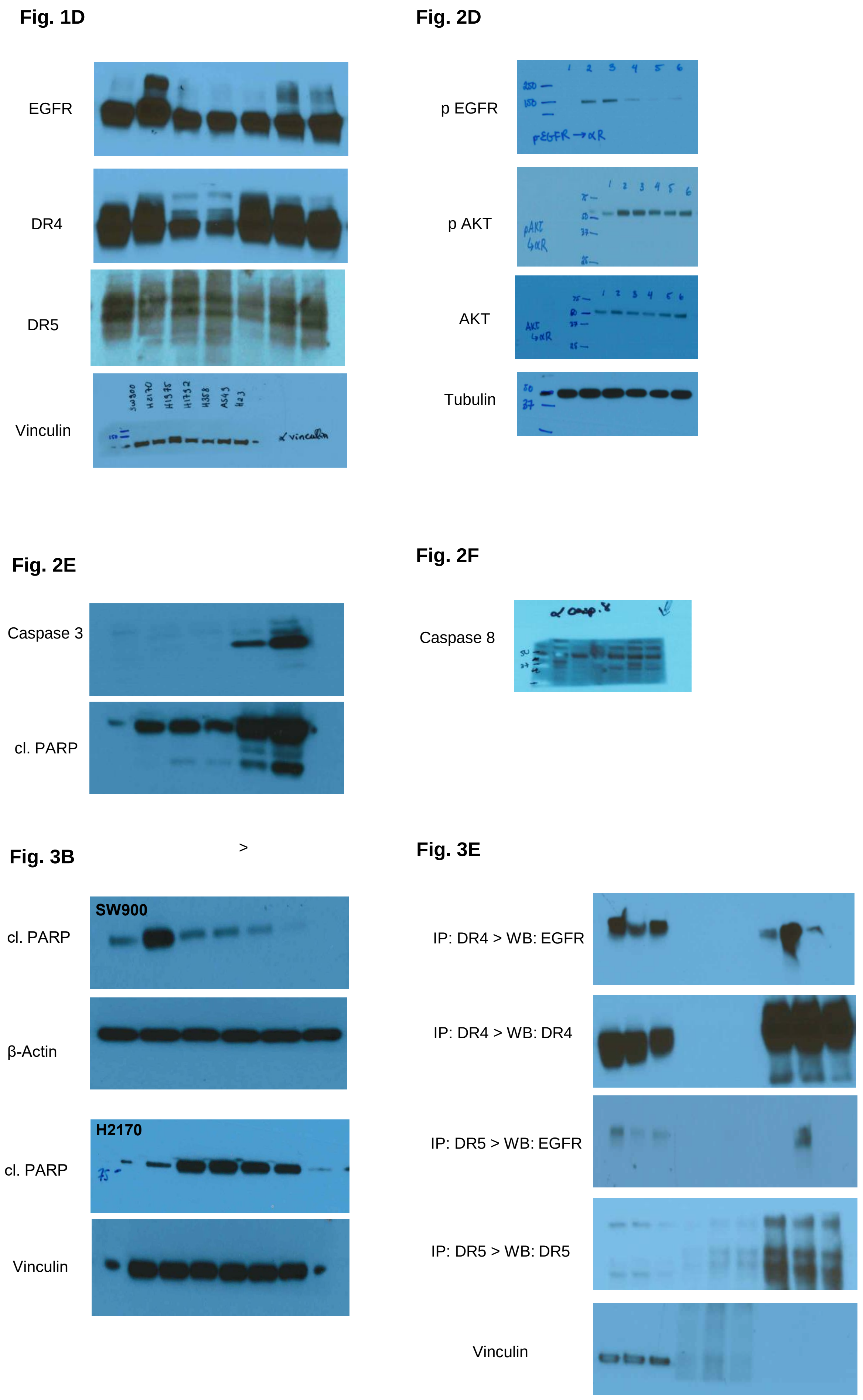

Figure S14. Related to Figure 1, Figure 2, and Figure 3. Unprocessed western blots.
