# Supplementary material for: Fate and Efficacy of Engineered Allogeneic Stem Cells Targeting Cell Death and Proliferation Pathways in Primary and Brain Metastatic Lung Cancer
Source: Stem Cells Transl Med. 2023 Jun 13;12(7):444–58. doi: 10.1093/stcltm/szad033 (PMC10346421; doi:10.1093/stcltm/szad033)
Supplement: szad033_suppl_Supplementary_Material [file szad033_suppl_supplementary_material.docx]

**Supplementary Material:**

**Material and Methods**

**Cell Lines and Cell Culture:** SW900, H2170, H358, A549, and H23 cell lines were kindly provided by Dr. Andrew Tilston-Lunel from Bob Varelas laboratory (Boston University, MA, USA), H1975 and H1792 were kind gifts from Dr. Ichiro Nakano laboratory (University of Alabama, Birmingham, USA), and PC9-BrM3 was kindly given by Dr. Adrienne Boire laboratory (Memorial Sloan Kettering Cancer Center, New York, USA). HEK293 cells together with all of the abovementioned cell lines were maintained in either Dulbecco’s Modified Eagle’s Medium (DMEM) or RPMI 1640 (Gibco) supplemented with 10% fetal bovine serum (Gibco) and antibiotics (100 units/ml penicillin and 100 μg/ml Streptomycin) (Gibco). Bone marrow-derived mouse and human mesenchymal stem cell (mMSC) lines were kindly obtained from Dr. Darwin Prockop (University of Texas), and cultured in DMEM supplemented with 1x MEM non-essential aminoacids (Gibco), 1% L-glutamine (Gibco), 100 units/ml penicillin and 100 μg/ml Streptomycin (Gibco), and 15% fetal bovine serum (Gibco). SW900-BM cell line was created as follows: female nude mice (6 to 8 weeks of age, 20 to 25 g, Envigo) were immobilized into a stereotactic frame (Stoelting). SW900 cells (1x10^5^ in 5uL) were injected into the mice brain parenchyma. Tumor growth was monitored through bioluminescence. Upon reaching an end point (appearance of neurological symptoms), animals were euthanized. Tumor bearing-brains were then extracted, partially homogenized, and digested in a collagenase solution in DMEM, extruded through a 19G needle, and strained with a 70µm cell strainer. After multiple centrifugations, the explant culture was maintained in DMEM 10% FBS. All cell lines were maintained at 37^0^C in a humidified atmosphere of 5% CO2 (v/v). All experiments were carried out on cells grown to 70-80% confluency.

**Engineering of E_V_DR_L_ and E_S_DR_L_ lentiviral vectors:** E_VHH_DR_L_ (E_V_DR_L_) consists of EGFR specific VHH domain (VH) fused to the extracellular domain of TRAIL (DR_L_) via a linker sequence and an isoleucine zipper. Specifically, the cDNA encoding extracellular domain of DR_L_ and an 18-aa linker was PCR amplified using forward primer and a reverse primer introducing an XhoI site. The PCR fragment was directionally inserted into EcoRV-XhoI digested LV-V_H_, resulting in LV-E_V_DR_L_. To create E_S_DR_L_ lentiviral construct, V_H_ domain in LV-E_V_DR_L_ was replaced by cDNA encoding EGFR ScFv, which was PCR amplified using forward primer and a reverse primer introducing a Nhe1 and EvoRV respectively. The remaining constructs used namely, E_V_, DR_L_, or GFP were previously described (28, 37). To obtain conditioned media (CM) containing different therapeutic proteins, lentiviral plasmid vectors coding for E_V_, DR_L_, E_V_DR_L_, and E_S_DR_L_ were transfected into 293T cells. Medium was changed the next day, collected 40 h after transfection, concentrated using centrifugal filter (#UFC901024, MilliporeSigma), and stored at −80 °C until future use.

**Lentiviral packaging and engineering of stable cell lines:** Lentiviral packaging was performed according to standard protocols. Briefly, HEK293T cells were co-transfected by using CaCl_2_ with packaging plasmids VSVG, CMV-Δ, and lentiviral vectors. Supernatants were collected 48h and 72h after transfection, and cells were transduced with 1.5 mL of viral supernatant (6-well plate) containing protamine sulfate (2 μg/mL). After 48h, transduced cells were selected with puromycin (1-2 μg/mL) and this selection maintained for 72h. The following diagnostic lentiviral vectors were used in this study: LV-Pico2-Fluc-mCherry bearing firefly luciferase (Fluc) and mCherry (FmC), LV-Pico2-Rluc-mCherry bearing *Renilla* luciferase (Rluc) and mCherry (RmC), LV-Pico2-Fluc-GFP (GFl), LV-Pico2-Rluc-GFP (GRl). Successful transduction was confirmed by fluorescence microscopy.

**Antibodies and reagents:** The following antibodies are available commercially: anti-β-Actin (#4970, 1:1000); anti-phospho-AKT (Ser473) (#4060, 1:1000); anti-AKT (#9272, 1:1000); anti-Caspase 8 (#9746, 1:1000); anti-cleaved Caspase 3 (#9661, 1:1000); anti-EGFR (#2646, 1:1000); anti-phospho-EGFR (Tyr1068), (#3777, 1:1000); anti-cleaved PARP (#9541, 1:1000); anti-phospho-p44/42MAPK(ERK1/2) (Thr202/Tyr204) (#9101, 1:1000); anti-p-44/42MAPK(ERK1/2) (#9102, 1:1000); HRP goat anti-rabbit secondary antibody (#7074, 1:2500) (Cell Signaling Technology); HRP goat anti-mouse secondary antibody (#ab205719, 1:2500) (Abcam); anti-α Tubulin (#T5168, 1:4000); anti-Vinculin (#V4505, 1:2000) (Sigma); anti-DR4 (#1139, 1:1000); anti-DR5 (#2019, 1:1000) (ProSci); anti-DR4 (#sc-32255, 1:1000), anti-DR5 (#sc-166624, 1:1000); Normal mouse IgG (#sc-2025) (Santa Cruz). *For immunofluorescence* the following antibodies were used at the stated concentrations: anti-CD31 (#ab28364, 1:200) (Abcam); anti-Ki-67 (#180191Z, 1:200) (Invitrogen); anti-cleaved Caspase 3 (#9661, 1:400) (Cell Signaling); Alexa Fluor anti-rabbit 405 (#A-31556, 4 µg/mL); Alexa Fluor anti-rabbit 488 (#A-11008, 4 µg/mL); Alexa Flour anti-rabbit 647 (#A-21244, 4 µg/mL) (ThermoFisher). *For IHC* the following antibodies were used at the stated concentrations: anti-EGFR (#D38B1, 1:50) (Cell Signaling); anti-DR4 (#1139, 1:100), anti-DR5 (#2019, 1:50) (ProSci). For flow cytometry the following antibodies were used at the stated concentrations: PE anti-human DR4 (#12-6644-42, 5 µl/test), PE anti-DR5 (#12-9908-425, 5 µl/test), PE mouse IgG isotype (#12-4714-42, 5 µl/test) (Invitrogen), PE anti-EGFR (#352903, 5 µl/1x10^6^ cells) (BioLegend).

Cetuximab (ImClone Systems Inc.) and human recombinant EGF (R&D Systems) were used at indicated concentrations according to manufacturer’s instructions.

**Immunoblot Analysis:** Cells were lysed post-treatment in a nonidet P-40 buffer (Boston BioProducts) supplemented with protease inhibitor cocktail (1:100) (Thermo or Roche) and phosphatase inhibitors (1:100) (Phosphatase Inhibitor Cocktail 2,3 from Sigma-Aldrich). Cells were scraped into tubes, left on ice for approximately 15 minutes and centrifuged at 4°C, 13,000 g for 15 minutes. Protein concentrations were determined using a Bio-Rad DCTM Protein Assay Kit. Protein lysates (25 μg to 60 μg) were run on an SDS/10% polyacrylamide gel and transferred onto PVDF, Immobilon-P membranes transfer membrane (Merck Millipore). To prevent nonspecific binding, membranes were incubated in blocking buffer (5% skimmed dried milk, 33.3 mM Tris-HCl, 16.68 mM Tris base, 138 mM NaCl, 2.7 mM KCl, 0.1% Tween-20) with agitation for 1h at room temperature, followed by immediate incubation with specific antibodies diluted in either 5% BSA or blocking buffer, overnight. Membranes were then washed three times in washing buffer (33.3 mM Tris-HCl, 16.68 mM Tris base; 138 mM NaCl; 2.7 mM KCl; 0.1% Tween-20), incubated for 1h at room temperature with goat anti-mouse HRP-conjugated antibody (sc-2005; 1:10000) or Protein A-HRP linked (NA9120V; 1: 2000; GE Healthcare) and protein expression was detected by chemiluminescence using ECL (ThermoFisher).

**Immunoprecipitation:** Cell lysates were collected post-treatment and proteins were isolated and quantified as described above. One mg of protein was immunoprecipitated with Protein A/G resins (St. Cruz Biotechnologies) and indicated antibody with gentle rotation at 4°C, overnight. Immunoprecipitates were washed 4 times in RIPA buffer, and bound proteins were dissociated in 25μL of 1x loading dye (25 mM Tris-HCl pH 6.8, 4% SDS, 5% glycerol, bromophenol blue). Samples were then centrifuged at 4°C, 13,000 g for 2 minutes, and the supernatant collected. Eluted proteins were separated on SDS/10% polyacrylamide gel and transferred onto Immobilon-P membranes (*vide* Immunoblotting).

**Cell viability and Caspase Assays:** Unless otherwise stated, 10,000 tumor cells were plated in 96-well plates and left incubating overnight (*o.n*.) at 37^0^C in a humidified atmosphere of 5% CO_2_ (v/v). On the following day, cells were treated at different concentrations with E_V_, E_s_DR_L_, DR_L_ or E_V_DR_L_ conditioned medium for 24h, 48h, or 72h. Cell viability for non-FLuc expressing cells was determined by reading the luminescence signal generated after cell lysis, proportional to the amount of the ATP present in live cells (CellTiterGlo, Promega). Caspase 3/7 and Caspase 9 activities were measured by using a DEVD-aminoluciferin substrate (Promega) according to manufacturer’s instructions. Plates were read using a spectrophotometer microplate reader set to 750 nm (GloMax-Multi Detection System, Promega).

**Flow cytometry:** Tumor cells (at least 0.5 x 10^5^ cells/per condition) were resuspended in PBS, centrifuged, resuspended in FACS stain buffer (BD Biosciences) and stained with PE-conjugated anti-EGFR, -DR4 or –DR5 monoclonal antibodies for 1h at 4ºC. Cells stained with PE-conjugated isotype specific IgG only were used as negative controls. Samples were analyzed on a FACS Aria II (BD Biosciences) cell sorter or on a LSRII flow cytometer (BD Biosciences) and assessed using FlowJo software (Tree Star, Ashland, OR). Where possible, a minimum of 10 000 events were collected for analysis.

**Enzyme-linked Immunosorbent Assay (ELISA):** Quantitative measurement of E_V_DR_L_ or DR_L_ released by MSC was determined by using a DR_L_ antigen capture ELISA (Enzyme-linked Immunosorbent Assay) kit (Abcam), according to manufacturer’s instructions.

**Modeling EGFR-E_V_-DR_L_-DR5 complex:** To build a model of the EGFR-E_V_-DR_L_-DR5 complex, we used the experimental crystal structures for each component that had a structure in the protein data bank (EGFR, E_VH1_, E_VH2_, DR_L_ and DR5). We then assembled pairwise complexes using the known relative orientation of various pairs of proteins in experimental complex structures as follows: EGFR- E_VH1_ from PDB ID 4KRL, EGFR- E_VH2_ from 4KRP, DR_L_ -DR5 from 1D0G. As in the crystal structure 4KRP, only partial structure of EGFR was solved, EGFR regions of 4KRL and 4KRP were superposed to conserve the EGFR structure and the E_VH_ interfaces. We then modeled missing residues of DR_L_ in 1D0G using Robetta (76). We manually positioned the EGFR-EV complex and the DRL-DR5 complex such that the transmembrane (TM) region of each were pointed in a similar direction at a distance that could be reasonably connected by the leucine zipper and linkers. The linkers and the leucine zipper were modelled using Rosetta’s *remodel* protocol (77). Finally, we identified the transmembrane regions of EGFR and DR5 using OCTOPUS and modeled those regions as ideal helices using Rosetta’s *helix_from_sequence* protocol, manually embedded the transmembrane regions in the membrane, and modeled the linkers to the transmembrane helices using Rosetta *remodel* (77). The picture of the membrane in Fig. 3C was created using NAMD and superposed manually on the complex model in PyMOL. (The PyMOL Molecular Graphics System, Version 2.0 Schrödinger, LLC).

**Coculture and GCV treatments**

hMSC-E_V_DR_L_-TK, at different cell ratios, were co-cultured with SW900-BM-FmC (2x10^3^ cells per well) in 96-well plate, and the relative number of SW900-BM-FmC was determined by Fluc luminescence. For analysis of the in vitro effect of GCV in hMSC-E_V_DR_L_-TK, cells were treated with different concentrations of GCV for 48h, and the relative cell viability determined by CellTiter-Glo (Promega)

***In vivo* imaging:** Tumor growth over time in mice tumor models was determined by bioluminescence imaging (BLI) of either FLuc or RLuc-engineered implanted cells using a PerkinElmer IVIS Lumina system. FLuc signal was determined 7 minutes after intraperitoneal injection of D-luciferin (PerkinElmer) and *Renilla* luciferase signal determined 1 minute after intravenous injection of Coelenterazine (PerkinElmer).

**Tissue processing:** For lung harvesting mice were anesthetized with ketamine/xylazine. Next, the chest cavity was exposed and the animals perfused with PBS followed by 4% paraformaldehyde. Lungs were then inflated through tracheal infiltration with a 1:1 OCT/PBS, harvested, cryoprotected in 30% sucrose for 24h at 4^0^C, and frozen in vinyl mold surrounded with OCT. Tissues were kept at -80^0^C until required. Subsequently, lungs were coronally sectioned into 8 μm sections by using a sliding microtome (#Micron^TM^ HM550 Cryostat, Thermo Scientific).

**Histological analyses:** Lung sections were washed in PBS and mounted with aqueous mounting medium (Vector Laboratories) and visualized under fluorescence microscope. For H&E staining, lung frozen coronal sections were stained with hematoxylin, counterstained with eosin Y, dehydrated in 95% and 100% ethanol, and mounted with xylene-based mounting medium (Cytoseal XYL, Thermofisher Scientific). Immunohistochemistry was performed using the Vectastain ABC kit (Vector) on 8 μm cut lung-frozen sections. Briefly, endogenous peroxidase was quenched using 3%H_2_O_2_ in methanol. Sections were blocked for 1h in PBS containing 1% BSA, 0.1% Tween 20 and 5% normal goat serum in a humidified chamber at room temperature. Sections were incubated with indicated primary antibodies diluted in PBS supplemented with 1% BSA, 0.1% Tween 20 overnight at 4^0^C. The following day, sections were extensively washed in PBS containing 1% BSA, 0.1% Tween 20 and incubated with appropriated secondary antibody for 30 minutes at room temperature. After extensive washing, 3,3’-diaminobenzidine tetrahydrochloride (DAB) was used for color development and nuclei were counterstained with hematoxylin as per the manufacturer’s recommendations. Sections were then dehydrated in 95% and 100% ethanol, cleared and mounted with xylene-based mounting medium for microscope evaluation (Cytoseal XYL, Thermofisher Scientific).

**Immunofluorescence:** For immunofluorescence staining of frozen coronal sections of the lung were incubated in blocking and permeabilization buffer (5% normal goat serum, 0.2% Triton-X in 50 mM PBS pH=7.4) for 1 hour at room temperature followed by overnight incubation with indicated primary antibodies diluted in 5% normal goat serum in 50 mM PBS. After five PBS washes, tissue sections were incubated with Alexa Fluor anti-rabbit 405 (4 µg/mL), Alexa Fluor anti-rabbit 488 (4 µg/mL) or Alexa Flour anti-rabbit 647 (4 µg/mL) (ThermoFisher) for 1.5 h at room temperature. After five PBS washes, sections were mounted in Vectashield antifade mounting medium with DAPI (#H-1200; Vector Laboratories) and examined under fluorescence microscope or using confocal microscopy (LSM 780; Carl Zeiss; Plan Neofluar 63x/1.3 NA Korr differential interference contrast M27 objective in water) at room temperature. Digitalized images were assembled using ZEN 2011 (64 bit) (Carl Zeiss) and ImageJ softwares.
